# A Bioinspired Manganese‐Organic Framework Ameliorates Ischemic Stroke through its Intrinsic Nanozyme Activity and Upregulating Endogenous Antioxidant Enzymes

**DOI:** 10.1002/advs.202206854

**Published:** 2023-05-02

**Authors:** Jian Wang, Yang Wang, Xiakeerzhati Xiaohalati, Qiangfei Su, Jingwei Liu, Bo Cai, Wen Yang, Zheng Wang, Lin Wang

**Affiliations:** ^1^ Department of Clinical Laboratory Union Hospital Tongji Medical College Huazhong University of Science and Technology Wuhan 430022 P. R. China; ^2^ Hubei Key Laboratory of Regenerative Medicine and Multi‐disciplinary Translational Research Research Center for Tissue Engineering and Regenerative Medicine Union Hospital Tongji Medical College Huazhong University of Science and Technology Wuhan 430022 P. R. China; ^3^ Department of Gastrointestinal Surgery Union Hospital Tongji Medical College Huazhong University of Science and Technology Wuhan 430022 P. R. China

**Keywords:** antioxidant enzyme, bioinspired nanozyme, metal‐organic frameworks (MOFs), reactive oxygen species (ROS), stroke treatment

## Abstract

Following stroke, oxidative stress induced by reactive oxygen species (ROS) aggravates neuronal damage and enlarges ischemic penumbra, which is devastating to stroke patients. Nanozyme‐based antioxidants are emerging to treat stroke through scavenging excessive ROS. However, most of nanozymes cannot efficiently scavenge ROS in neuronal cytosol and mitochondria, due to low‐uptake abilities of neurons and barriers of organelle membranes, significantly limiting nanozymes’ neuroprotective effects. To overcome this limitation, a manganese‐organic framework modified with polydopamine (pDA‐MNOF), capable of not only mimicking catalytic activities of natural SOD2's catalytic domain but also upregulating two endogenous antioxidant enzymes in neurons is fabricated. With such a dual anti‐ROS effect, this nanozyme robustly decreases cellular ROS and effectively protects them from ROS‐induced injury. STAT‐3 signaling is found to play a vital role in pDA‐MNOF activating the two antioxidant enzymes, HO1 and SOD2. In vivo pDA‐MNOF treatment significantly improves the survival of middle cerebral artery occlusion (MCAo) mice by reducing infarct volume and more importantly, promotes animal behavioral recovery. Further, pDA‐MNOF activates vascular endothelial growth factor expression, a downstream target of STAT3 signaling, thus enhancing angiogenesis. Taken together, the biochemical, cell‐biological, and animal‐level behavioral data demonstrate the potentiality of pDA‐MNOF as a dual ROS‐scavenging agent for stroke treatment.

## Graphical abstract

By mimicking the catalytic domains of natural antioxidant enzymes, a de novo metal‐organic framework modified with polydopamine is fabricated. It not only possesses superoxide dismutase‐like nanozyme activities but also biologically upregulates two endogenous antioxidant enzymes (HO1 and SOD2) through STAT3 signaling, effectively scavenging excessive reactive oxygen species and thereby powerfully salvaging neurons from ischemic insult poststroke.

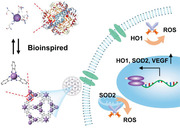

## 1 Introduction

Stroke is the second leading cause of death and the most common cause of permanent disability,^[^ ^1^, ^2^ ^]^ bringing huge burdens to public health. Currently, thrombolysis and neurothrombectomy are two clinically available treatments to rescue neurons within ischemic and hypoxic cores.^[^ ^3^ ^]^ However, the majority of stroke patients fails to benefit from these therapies due to their narrow treatment window (4.5 to 6 h after onset).^[^ ^4^, ^5^ ^]^ For these patients, the main aim of stroke treatment is to salvage ischemic penumbra adjacent to infarcted cores to minimize the size of eventually formed infarct and ameliorate neurological dysfunction.^[^ ^6^ ^]^ One of the predominant pathological characteristics of ischemic penumbra is excessive reactive oxygen species (ROS) generated from ischemia and reperfusion.^[^ ^7^ ^]^ Among these ROS, superoxide radical (O_2_^●−)^ is the main initial and vital active radicals, which participates in the generation of secondary reactive oxygen and nitrogen species (RONS) including hydroxyl radical (^●^OH) and peroxynitrite (ONOO‐), further aggravating oxidative injury.^[^ ^8^ ^]^ Superoxide radical and these generated secondary RONS overwhelm intracellular antioxidant enzyme system and even subversively react with nucleic acids, lipids, and proteins, ultimately leading to irreversible neuronal death.^[^ ^6^, ^9^ ^]^ Therefore, effective approaches scavenging excessive ROS to rescue neurons of penumbra are highly desired.

Numerous efforts have been directed to eliminate ROS in ischemic penumbra for stroke treatment. Antioxidant agents, such as edaravone, disufenton sodium (i.e., NXY‐059), and ebselen,^[^ ^10^, ^11^, ^12^ ^]^ often deplete ROS by consuming themselves,^[^ ^13^ ^]^ which however requires repeated administration and thus is of low efficiency. In contrast, natural antioxidant enzymes, such as superoxide dismutase (SOD) and catalase (CAT), remain constant as they catalyze anti‐ROS cascade reactions. Thus, a potential anti‐ROS strategy could be to utilize exogenously engineered SOD and CAT,^[^ ^14^, ^15^, ^16^ ^]^ which nevertheless is of high cost, low stability, and potential immunogenicity of natural enzymes.^[^ ^17^ ^]^ Nanozymes, a type of nanomaterials with enzyme activities, are capable of overcoming these aforementioned limitations and has been becoming a promising therapeutic alternative for stroke treatment.^[^ ^18^ ^]^ A number of nanozymes have been explored to tackle excessive ROS in stroke treatment, e.g., ceria nanoparticles with ROS‐scavenging activities for effectively protecting neurons,^[^ ^19^ ^]^ Prussian blue‐based nanozymes protecting neurons against ischemic stroke through attenuating oxidative stress,^[^ ^20^, ^21^ ^]^ and nanozymes with multiple catalytic activities ameliorating oxidative injury.^[^ ^22^, ^23^, ^24^ ^]^ Indeed, these nanozymes after being delivered into brain effectively lower ROS in neurons’ local microenvironment, including blood, interstitial fluids, and cerebrospinal fluids, but not within neurons. This is because of these nanozymes’ low uptake by neurons due to the barriers of organelle membranes. Meanwhile, imbalance of redox homeostasis resulting from overwhelming ROS generation deeply damages cellular biosynthetic progress, interrupting timely replenishment of endogenous antioxidant enzymes in neurons.

Metal‐organic framework (MOF) has drawn extensive attention due to their multiple active sites, high specific surface areas, and ease of large‐scale production.^[^ ^25^ ^]^ Inspired by the thought of functionally and chemically imitating the catalytic domains of natural antioxidant enzymes, we set to fabricate a de novo MOF with “nanozyme‐like” catalytic activities.^[^ ^26^ ^]^ Herein, we reported an MNOF with a manganese metal center coordinated by six nitrogen atoms of 2,3,6,7,10,11‐hexaiminotriphenylene (HATP), similar to the catalytic domain of natural SOD2, whose catalytic domains contained a manganese metal fixed by three histidine and one aspartate.^[^ ^27^ ^]^ Notably, this MNOF not only possessed SOD‐like nanozyme activities but also biologically upregulated two endogenous antioxidant enzymes in neuronal cells, HO1 (mainly distributed within cytosol) and SOD2 (within mitochondria). Activation of endogenous antioxidant enzymes compensated the inability of the nanozymes to remove intracellular ROS, effectively protecting neurons from ROS‐induced injury. To the best of our knowledge, few studies have reported this type of nanozymes with dual (chemical and biological) ROS‐scavenging effects on salvaging neurons in ischemic penumbra.

**Scheme 1:**
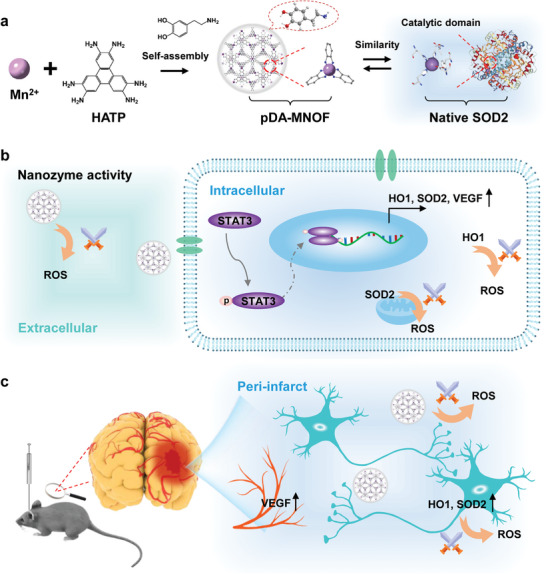
Schematics of an SOD2‐inspired manganese‐organic framework to effectively scavenge ROS for ameliorating ischemic stroke. a) Schematics of fabrication of pDA‐MNOF that possessed a bioinspired structure mimicking the catalytic domain of native SOD2. b) Schematics of pDA‐MNOF scavenges extracellular ROS with nanozyme activities, while reducing intracellular ROS through upregulating endogenous antioxidant enzymes, HO1 and SOD2 via activating STAT3 signaling. c) Schematics of pDA‐MNOF‐based therapy ameliorating ischemic stroke through dual activities: ROS‐scavenging activity and angiogenesis‐promoting activity.

In this study, we demonstrated that an MOF modified with polydopamine (pDA‐MNOF) possessed SOD‐like catalytic activities with the capability of activating two endogenous antioxidant enzymes (**Scheme** **1**). STAT3 signaling was found to play a vital role in mediating pDA‐MNOF's upregulating HO1 and SOD2. In a preclinical stroke animal model (middle cerebral artery occlusion, MCAo), the therapeutic effect of pDA‐MNOF was significant as evidenced by reduced infarct volume, enhanced neuron survival, and improved recovery of animal‐level behaviors. This nanozyme exhibited excellent tissue compatibility and in vivo biosafety. This study not only provided a bioinspired pDA‐MNOF with unique dual ROS‐scavenging activities, but also demonstrated its in vivo therapeutic efficacy in treating stroke.

## 2 Results and Discussion

### 2.1 Preparation and Characterization of MNOF

**Figure 1:**
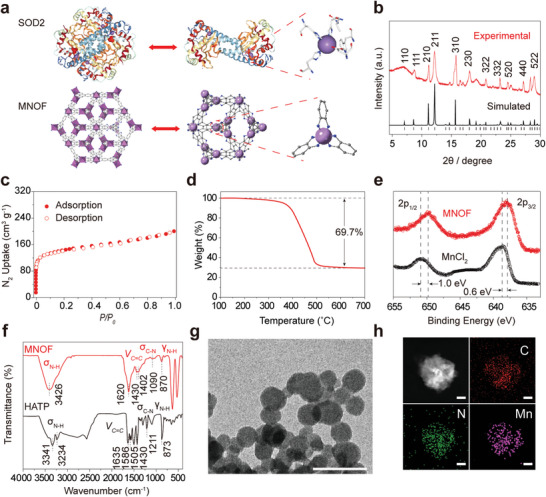
Characterization of MNOF. a) Structure of native SOD2 enzyme and MNOF. Purple balls represent manganese atoms. b) PXRD pattern of the MNOF (red) and the simulated one (black). c) N_2_ isotherm of MNOF at 77 K. Filled and open symbols represent adsorption and desorption balance, respectively. d) TGA analysis of MONF. e) XPS spectra analysis of MNOF and MnCl_2_. f) FTIR spectra of MNOF and HATP linker. g) HR‐TEM images of MNOF. Scale bar, 100 nm. h) Elemental maps of MNOF sample revealed by EDS analysis. The elements are given at the top right of each colored map. Scale bar, 10 nm.

The MNOF was prepared as the previously reported method with some modifications.^[^ ^28^ ^]^ The bioinspired MNOF possessed multiple active centers consisting of a manganese and six nitrogen atoms (**Figure** **1**a), which was structurally resembled the actively catalytic domains of natural SOD2 where a manganese atom was coordinated by three histidine and one aspartate.^[^ ^27^ ^]^ The consistent binding energy of manganese between MNOF and natural SOD2 further supported the similarity of their catalytic domains (Figure S1, Supporting Information). Powder X‐ray diffraction (PXRD) of the MNOF matched well with the simulated one (Figure 1b), revealing the successful synthesis of this framework. The porosity of MNOF was high as N_2_ adsorption assay at 77 K showed that the Brunauer–Emmett–Teller areas of the framework reached 510 m^2^ g^−1^ (Figure 1c). Thermogravimetric analysis (TGA) showed 69.7% of weight loss under the continuous air flow (Figure 1d), verifying the existence of organic components in MNOF. X‐ray photoelectron spectroscopy (XPS) analysis showed that the 3p_3/2_ and 3p_1/2_ orbitals of MNOF were shifted by 0.6 and 1.0 eV, respectively, compared to the original MnCl_2_ salt (Figure 1e), suggesting the successful coordination between Mn atoms and HATP linkers. Further supporting this notion, Fourier transform infrared spectra (FTIR) analysis revealed the shifting of characteristic peaks: the stretching vibration of *ν*_C=C_ in the benzene ring at 1635, 1586, 1505, and 1430 cm^−1^ in HATP linker shifting to be within the range of 1620–1402 cm^−1^ in MNOF (Figure 1f). The associated stretching vibration of N—H—N hydrogen bonds that showed two peaks at 3341 and 3234 cm^−1^ in the organic linker became one peak at 3426 cm^−1^ in MNOF (Figure 1f), suggesting the disassociation of N—H—N hydrogen bonds during MNOF construction. High‐resolution transmission electron microscopy (HR‐TEM) revealed MNOF nearly spherical morphology with the size of ≈50 nm (Figure 1g). In addition, the energy dispersive X‐ray spectroscopy (EDS) analysis on MNOF showed that manganese, nitrogen, and carbon species were uniformly dispersed throughout the framework (Figure 1h).

### 2.2 In Vitro SOD‐Like Activity of MNOF and Their O_2_^●−^ Scavenging Mechanism

**Figure 2:**
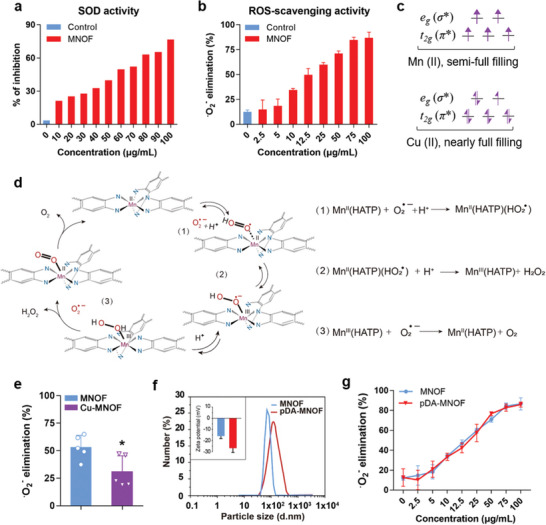
The activity and mechanism of MNOF scavenging O_2_^●−^. a) SOD‐like catalytic activity of MNOF at different concentrations. b) O_2_^●−^ scavenging effect of MNOF at a series of concentrations (*n* = 6). c) 3d electron occupancy of *t*_2g_ (π*) and *e*_g_ (σ*) antibonding orbitals associated with the transition metal for Mn (II) and Cu (II). d) The suggested mechanism of pDA‐MNOF scavenging superoxide radical. e) Superoxide radical activity of MNOF and Cu‐MNOF at 12.5 µg mL^−1^ (*n* = 5). Data were presented with mean ± s.d.; *, *p* < 0.05; *T*‐test. f) DLS analysis of MNOF and pDA‐MNOF. Inset: zeta potential analysis of MNOF (blue) and pDA‐MNOF (red). g) Superoxide radical scavenging activity of MNOF and pDA‐MNOF at various concentrations (*n* = 6).

The SOD‐like activity of MNOF was determined using the commercial SOD detection kit as previously described.^[^ ^29^ ^]^ Briefly, the water‐soluble tetrazolium salt (WST‐1) was used to monitor O_2_^●−^ generated from the mixture of xanthine oxidase (XO) and xanthine (X), while this process was inhibited in the presence of nanozymes with SOD‐like activity. Higher inhibition ratios indicate stronger SOD‐like activities. With the concentrations increasing, the MNOF showed gradually enhanced inhibition ratios (**Figure** **2**a), indicating its dose‐dependent SOD‐like activity. This concentration‐linked scavenging activity was further confirmed by the analysis using nitrotetrazolium blue chloride (NBT), a sensitive probe of O_2_^●−^ ROS (Figure 2b). The electron spin‐resonance spectroscopy (ESR) results further verified the O_2_^●−^ scavenging activities of this nanozyme (Figure S2, Supporting Information). The SOD‐like activities of MNOF could reach 58.3% of that of natural SOD (Figure S3a, Supporting Information), which was comparable to that of reported nanozymes,^[^ ^30^, ^31^, ^32^ ^]^ and given its advantages of easy availability, low cost, and good stability, the nanozyme holds great potential as an antioxidant for biomedical application. Additionally, no obvious catalytic activities of HO1, CAT, and GPX1 were detected in the MNOF (Figure S3b–d, Supporting Information), and this nanozyme failed to scavenge hydroxyl radical (^●^OH), singlet oxygen (^1^O_2_), hydrogen peroxide (H_2_O_2_), nitroxide radical (^●^NO), and DPPH radical (DPPH^●^) (Figures S3c and S4a–d, Supporting Information), indicating the specificity of MNOF catalytic activity.

The d‐electron population was an effective indicator for the catalytic activity of nanozymes.^[^ ^33^ ^]^ The d‐electron orbital of Mn (II) in MNOF was semi‐full filling (Figure 2c), facilitating the gain and loss of the electrons during the catalytic cycle. Herein, a catalytic cycle was proposed to elaborate chemical reactions during MNOF scavenging O_2_^●−^ (Figure 2d). In MNOF, a bivalent Mn ion fixed with four nitrogen‐atoms in an equatorial position and two nitrogen‐atoms in axial position formed an active catalytic domain (Figure 1a). It was reported that O_2_^●−^, as a Brønsted base, could capture a proton from aqueous solution to form HO_2_^●^.^[^ ^34^ ^]^ When the HO_2_^●^ replaced one of the nitrogens in the ligand and bound to the Mn (II) center, one electron was lost in d orbital with the Mn ion shifting from the bivalent to the trivalent state. Then, the associative intermediate was protonated successively to release hydrogen peroxide. With the un‐full filling state of d‐electron orbital, the Mn (III) complex accommodated an electron to bind another superoxide anion radical, generating oxygen and completing the catalytic cycle (Figure 2d). Additionally, the catalytic mechanism was also confirmed by the density functional theory (DFT) calculations (Figure S5a, Supporting Information). This facilitation of the gain and loss of electrons in Mn active center during the catalytic cycle indicated that the population of the d‐electrons significantly influenced nanozyme activity. To further examine this notion, we utilized Cu (II) ion to partly replace Mn (II) in MNOF to prepare a hybrid framework, termed as Cu‐MNOF (Figure S5b, Supporting Information). As the d‐electron orbital in the Cu (II) was nearly fully filled (Figure 2c), hardly gaining more electrons during the catalytic cycle, the nanozyme activity of Cu‐MNOF would be compromised. Supporting this, the Cu‐MNOF analyzed by NBT assay indeed showed a decreased catalytic activity (Figure 2e).

To improve the dispersion stability and cyto‐compatibility, we modified the surfaces of MNOF with polydopamine as previously reported^[^ ^35^ ^]^ and the resultant was termed as pDA‐MNOF (Figure S6a,b, Supporting Information). The pDA‐MNOF exhibited spherical morphology with slightly increased sizes after modifications (Figure S6c, Supporting Information). Dynamic light scattering (DLS) analysis showed that the hydrodynamic diameter was ≈120 nm and zeta potential was ≈−26 mV for pDA‐MNOF (Figure 2f). Moreover, the TGA and elemental analysis further confirmed that pDA was successfully modified on the surfaces of MNOF (Figure S6d,e, Supporting Information). No significant difference was observed in superoxide radical elimination ratios between MNOF and pDA‐MNOF (Figure 2g), suggesting that polydopamine modification does not affect the SOD‐like activities of the nanozymes. Enhanced performance of pDA‐MNOF on the dispersion stability and cyto‐compatibility was confirmed by free settling experiment and CCK‐8 assay (Figure S7a,b, Supporting Information). Furthermore, the significantly improved dispersion and stability of pDA‐MNOF was also observed in other solvents, including distilled water, Dulbecco's modified Eagle medium (DMEM), and fetal bovine serum (FBS, Figures S8 and S9, Supporting Information).

### 2.3 pDA‐MNOF Biologically Upregulates SOD2 and HO1 Expression through STAT3 Signaling

**Figure 3:**
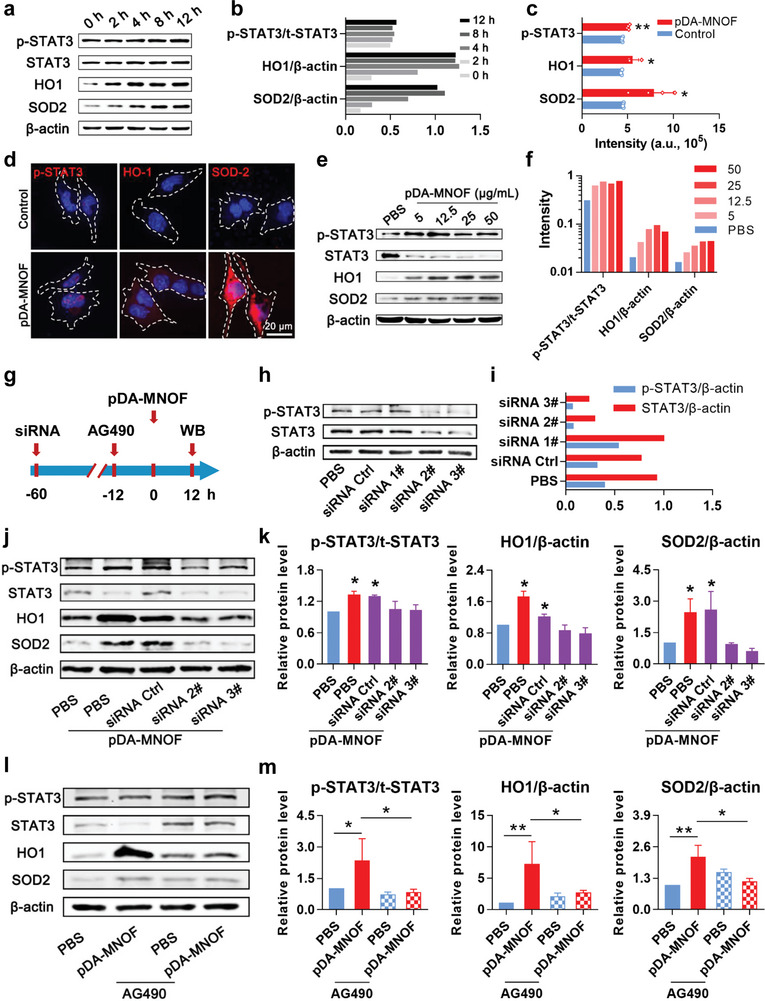
The effect of pDA‐MNOF on upregulating HO1 and SOD2 via STAT3 signaling. a) Western blot analysis of p‐STAT3, STAT3, HO1, and SOD2 expression in N2a cells treated with 12.5 µg mL^−1^ pDA‐MNOF for various time durations. b) Quantification of the band intensity ratios of p‐STAT3/STAT3, HO1/*β*‐actin, and SOD2/*β*‐actin in (a). t‐STAT3 indicated total proteins of p‐STAT3 and STAT3. c) Quantification of fluorescent intensity of p‐STAT3, HO1, and SOD2 in PBS or 12.5 µg mL^−1^ pDA‐MNOF‐treated N2a cells (*n* = 4). Data were presented with mean ± s.d.; *, *p* < 0.05; **, *p* < 0.01; *T*‐test. d) Representative images of immunofluorescent staining for p‐STAT3, HO‐1, and SOD‐2 (red) in indicated groups. Nuclei were stained in blue, and cell body was outlined with white dashed lines. Scale bar, 20 µm. e) Western blot analysis of p‐STAT3, STAT3, HO1, and SOD2 expression in N2a cells treated with different concentrations of pDA‐MNOF for 12 h. f) Quantification of the band intensity ratios of p‐STAT3/STAT3, HO1/*β*‐actin, and SOD2/*β*‐actin in (e). g) Experimental scheme for the RNA interference and pathway inhibition assays. h) Western blot analysis of p‐STAT3 and STAT3 in N2a cells treated with PBS, siRNA Control (siRNA Ctrl), siRNA 1#, siRNA 2#, and siRNA 3#. i) Quantification of the band intensity ratios of p‐STAT3/*β*‐actin and STAT3/*β*‐actin in (h). j) N2a cells were transfected with siRNA Ctrl, siRNA 2#, and siRNA 3#, followed by treatment with pDA‐MNOF; the lysates were analyzed with indicated antibodies. k) Quantification of the band intensity ratios of p‐STAT3/*β*‐actin, HO1/*β*‐actin, and SOD2/*β*‐actin in (j). Data were presented with mean ± s.d.; *, *p* < 0.05; ANOVA. l) N2a cells were treated with PBS or pDA‐MNOF, followed by treatment with AG490; the lysates were analyzed with indicated antibodies. m) Quantification of the band intensity ratios of p‐STAT3/STAT3, HO1/*β*‐actin, and SOD2/*β*‐actin in (l) (*n* = 3). Data were presented with mean ± s.d.; *, *p* < 0.05; **, *p* < 0.01; ANOVA.

Manganese (Mn) is an essential component of SOD2 (also named Mn‐SOD) and thus influences the assembly and expression of this antioxidant enzyme. Here, we determined whether pDA‐MNOF containing Mn influenced the expression of SOD2 and the other four typical endogenous antioxidant enzymes, including HO1, CAT, GPX1, and SOD1. Although no significant changes were detected in mRNA level of CAT, GPX1, and SOD1 in N2a cells after being treated with pDA‐MNOF for 12 h, the levels of two endogenous antioxidant enzymes, HO1 and SOD2, were significantly elevated (Figure S10a–e, Supporting Information). To further verify the effect of pDA‐MNOF on upregulating HO1 and SOD2, we treated N2a cells with pDA‐MNOF for different time durations and the protein levels of HO1 and SOD2 were drastically increased 2 h later and reached peak values (a 4.3‐fold and 6.3‐fold increase for HO1 and SOD2, respectively) 12 h after pDA‐MNOF treatment (**Figure** **3**a,b), suggesting the pDA‐MNOF's rapid and strong activation on these two antioxidant enzymes (Figure 3c,d) but not CAT (Figure S11a–d, Supporting Information). Such activation of HO1 and SOD2 induced by pDA‐MNOF was dose‐dependent (Figure 3e,f).

STAT3 signaling was reported to positively regulate SOD2 expression.^[^ ^36^ ^]^ A cluster of STAT3 signaling‐associated genes were elevated at mRNA levels after pDA‐MNOF treatment (Figure S12, Supporting Information). The ratio of phosphorylated STAT3 (p‐STAT3) to total STAT3 increased as the treatment time was extended (Figure 3a,b), indicating the progressive activation of STAT3 signaling. This type of STAT3 signaling activation was correlated with pDA‐MNOF doses (Figure 3e,f), suggesting that STAT3 signaling might be involved in pDA‐MNOF upregulating HO1 and SOD2. Given that p‐STAT3 homodimerizes and translocates into nuclei where it acts as a transcription factor to activate downstream target genes,^[^ ^37^ ^]^ we then investigated the nucleic distribution of p‐STAT3 using immunofluorescence staining assay and found the significantly increased p‐STAT3 in the nuclei of pDA‐MNOF‐treated N2a cells (Figure 3c,d), indicating the activation of STAT3 signaling.

To comprehensively study the role of STAT3 signaling in pDA‐MNOF activating HO1 and SOD2, the RNA interference (RNAi) and pathway inhibition assays were performed (Figure 3g). siRNA 2# and siRNA 3# capable of silencing p‐STAT3 and STAT3 expression were utilized for following RNAi experiments (Figure S13, Supporting Information and Figure 3h,i). The elevation of HO1 and SOD2 proteins in pDA‐MNOF‐treated N2a cells was abolished upon STAT3 silencing (Figure 3j,k), indicating the requirement of STAT3 signaling in mediating HO1 and SOD2 upregulation. Next, we utilized AG490, a STAT3 signaling inhibitor, to suppress the phosphorylation of STAT3, and found that pDA‐MNOF failed to upregulate proteins of HO1 and SOD2 in these AG490‐treated N2a cells (Figure 3l,m). Collectively, these results indicate that STAT3 signaling mediates pDA‐MNOF activating the two endogenous antioxidant enzymes.

To investigate whether the pDA‐MNOF could be internalized into neuronal cells, immunofluorescence staining of cytoskeleton and endoplasmic reticulum (ER), two sub‐organelle structures, was performed to test this nanozyme‐treated N2a cells (Figure S14a, Supporting Information). The granular pDA‐MNOF could be clearly identified in the differential interference contrast images (Figure S14b, Supporting Information). The analysis on nanozyme's localization in neuronal cells from 3D‐reconstructed images showed that the pDA‐MNOF was mainly colocalized with cytoskeleton rather than ER (Figure S14c, Supporting Information), indicating that this nanozyme is not internalized into the cytoplasm, but located on the cell membranes of N2a cells. Janus kinase (JAK), which was closely coupled with receptors residing in cell membranes, could phosphorylate STAT3 homodimerizes, thus initiating the activation of STAT3 signaling.^[^ ^38^, ^39^ ^]^ Based on this, we proposed that the pDA‐MNOF located on cell membranes might phosphorylate the JAK through binding to an unknown receptor and then activated STAT3 signaling, therefore upregulating the expression of intracellular SOD2 and HO1.

### 2.4 pDA‐MNOF Reduces Cellular ROS and Protects Neuronal Cells from OGD‐Induced Injury

**Figure 4:**
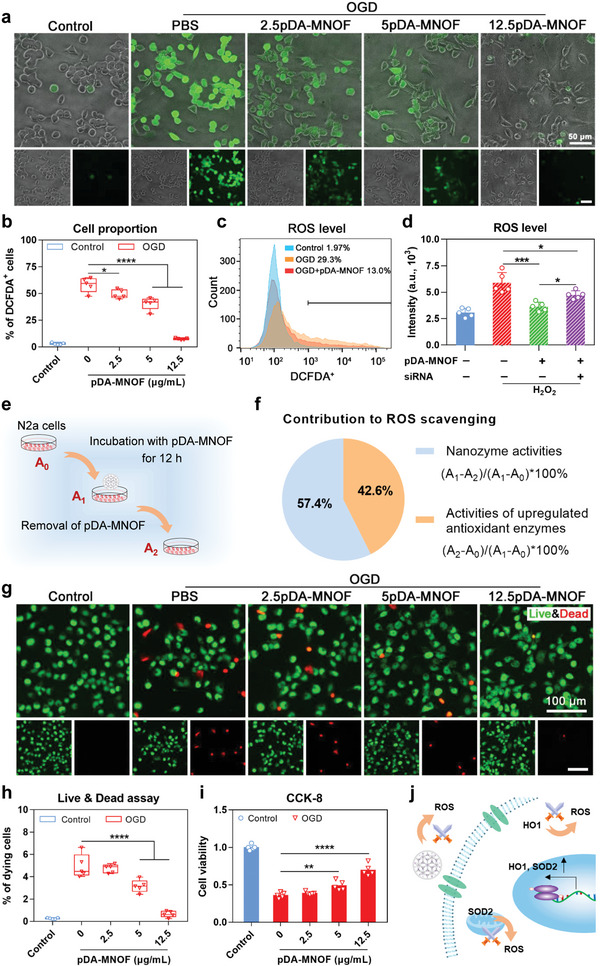
pDA‐MNOF protects neuronal cells against ROS‐induced injury. a) DCFH‐DA staining for detecting ROS in the PBS or pDA‐MNOF‐treated N2a cells after being treated with an 8 h OGD. N2a cells cultured in regular condition were set as the control. Scale bars, 50 µm. b) Quantification of percentages of DCFH‐DA positive (DCFDA^+^) cells in indicated groups in (a) (*n* = 5). Data were presented with mean ± s.d.; *, *p* < 0.05; ****, *p* < 0.0001; ANOVA. c) Flow cytometric analysis of DCFH‐DA positive cell proportions in the three groups. d) Fluorescence intensity detection in cell lysis of DCFH‐DA‐stained N2a cells in indicated groups (*n* = 5). Data were presented with mean ± s.d.; *, *p* < 0.05; ***, *p* < 0.001; ANOVA. e) Schematic illustration of determining the contribution ratios of two effects of pDA‐MNOF on scavenging ROS. Briefly, N2a cells were treated with PBS, or 12.5 µg mL^−1^ pDA‐MNOF for 12 h, and their SOD‐like activities were determined as A_0_ and A_1_, respectively. Cell lysis of pDA‐MNOF‐treated N2a cells was subject to ultracentrifugation for removing the residual pDA‐MNOF, and the SOD‐like activity was determined as A_3_. f) Quantification of contribution ratios of two effects of pDA‐MNOF on scavenging ROS. The formula was listed to the right. g) Live & Dead staining for PBS or pDA‐MNOF‐treated N2a cells after an 8 h OGD followed by a 16 h regular incubation. Scale bars, 100 µm. h) Quantification of percentages of dying cells in indicated groups in (g) (*n* = 5). Data were presented with mean ± s.d.; ****, *p* < 0.0001; ANOVA. i) Cell viability of N2a cells in indicated groups in CCK‐8 assay (*n* = 5). Data were presented with mean ± s.d.; **, *p* < 0.01; ****, *p* < 0.0001; ANOVA. j) Schematics of pDA‐MNOF utilizing nanozyme activity and upregulating endogenous HO1/SOD2, jointly scavenging ROS.

Given that pDA‐MNOF possessed SOD‐like activity and also biologically upregulated two endogenous antioxidant enzymes, HO1 and SOD2, we sought to determine whether the nanozymes were capable of decreasing ROS levels in neuronal cells. To test this, an oxygen‐glucose deprivation (OGD) in vitro model, which was widely used to mimic ROS production and ROS‐induced neuron death in vivo during stroke, was established. Under OGD, the percentage of ROS‐positive cells, positively stained by 2′,7′‐dichlorofluorescein diacetate (DCFH‐DA), was 58.2%, much higher than 3.0% of them in control (**Figure** **4**a,b), indicating the successful establishment of this model. After incubation with the increased concentrations of pDA‐MNOF, N2a cells under OGD showed the correspondingly reduced proportion of ROS‐positive cells (Figure 4a,b), indicating that pDA‐MNOF effectively reduces cellular ROS in a dose‐dependent manner. This cellular ROS scavenging effect of pDA‐MNOF was also evidenced by flow cytometric analyses (Figure 4c). Moreover, we constructed another H_2_O_2_ treated cell model to mimic ROS generation (Figure S15a,b, Supporting Information) and found that either MNOF or pDA‐MNOF could effectively decrease ROS levels (Figure S15c, Supporting Information). After specifically silencing the expression of HO1 and SOD2 (Figure S16a–f, Supporting Information), pDA‐MNOF's ROS‐scavenging activity was significantly decreased (Figure 4d), demonstrating that the upregulated HO1 and SOD2 play vital roles in pDA‐MNOF reducing cellular ROS. Thus, to determine individual contribution of either the SOD‐like nanozyme activity or upregulated HO1/SOD2 enzyme activity to ROS reduction in pDA‐MNOF‐treated neuronal cells, we removed pDA‐MNOF from cell lysate by ultracentrifugation and tested the difference in total ROS‐scavenging activity (A_1_−A_2_) (Figure 4e). The counterpart activity of upregulated HO1/SOD2 was calculated as the difference between A_2_ and A_0_ (control). By using these two formulas in Figure 4f, the contribution of SOD‐like nanozyme activity and upregulated HO1/SOD2 enzyme activity in pDA‐MNOF decreasing cellular ROS were determined to be 57.4% and 42.6%, respectively.

Next, we investigated whether pDA‐MNOF protected neuronal cells from ROS‐induced injury. Under OGD, 2.5 µg mL^−1^ pDA‐MNOF treatment had 4.7% dying cells, similar to that (4.6%) in phosphate‐buffered saline (PBS) control (Figure 4g,h), indicating pDA‐MNOF at low doses is not potent enough to protect neuronal cells. When its concentration went up to 5 and 12.5 µg mL^−1^, the dying cell proportion was decreased to be 3.2% and 0.7% (Figure 4g–i), respectively, confirming the neuroprotective activity of pDA‐MNOF. Additionally, the dose‐dependent neuroprotective activity of pDA‐MNOF was also verified in a H_2_O_2_‐induced cell injury model (Figure S15d, Supporting Information). Thus, these results comprehensively characterize the two effects of pDA‐MNOF on reducing cellular ROS levels, intrinsic chemical nanozyme activity, and biologically upregulated expression of HO1/SOD2 (Figure 4j), therefore collectively protecting neuronal cells from ROS‐induced injury.

### 2.5 pDA‐MNOF Reduces ROS Levels, Neuronal Death, and Infarct Volume In Vivo

**Figure 5:**
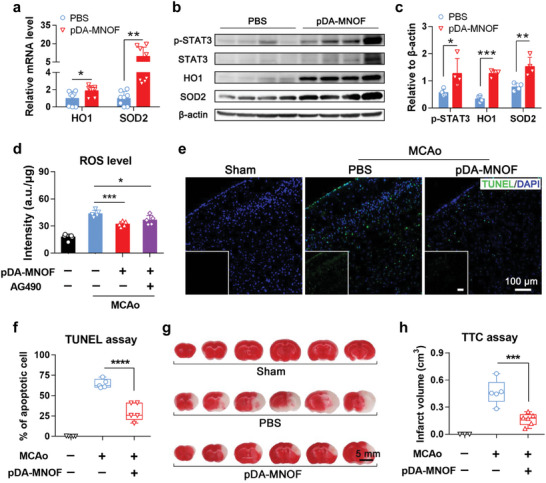
pDA‐MNOF upregulate in vivo HO1 and SOD2, and lowers ROS level, neuron death, and infarct volume. a) The mRNA levels of HO1 and SOD2 in stroke‐affected brain tissues of the MCAo mice receiving cerebral injection of PBS or pDA‐MNOF (*n* = 8). Data were presented with mean ± s.d.; *, *p* < 0.05; **, *p* < 0.01; *T*‐test. b) The protein expression of p‐STAT3, STAT3, HO1, and SOD2 in stroke‐affected brain of the MCAo mice receiving cerebral injection of PBS or pDA‐MNOF. c) Quantification of the band intensity ratios in (b) (*n* = 4). Data were presented with mean ± s.d.; *, *p* < 0.05; **, *p* < 0.01; ***, *p* < 0.001; *T*‐test. d) The ROS levels in stroke‐affected brain of the MCAo mice receiving PBS, pDA‐MNOF, or the combination of pDA‐MNOF and AG490 (STAT3 signaling inhibitor) (*n* = 5). The ROS level in brain of sham‐operated mice was set as the control. Data were presented with mean ± s.d.; *, *p* < 0.05; ***, *p* < 0.001; ANOVA. e) TUNEL (green) and DAPI (blue) staining in brain sections of the sham‐operated mice and the MCAo mice receiving PBS or pDA‐MNOF injection. Each image was reduced by 7.3‐fold and placed on the lower left corner. Scale bars, 100 µm. f) Quantification of percentages of TUNEL stained cells in (e) (*n* = 5). Data were presented with s.d.; ****, *p* < 0.0001 ANOVA. g) TTC staining in brain sections of sham‐operated mice and the MCAo mice receiving PBS or pDA‐MNOF treatment 24 h after receiving different treatments. Unaffected brain tissues were stained in red and ischemic brain tissues were indicated as white. Scale bar, 5 mm. h) Quantification of infarct volumes in (g). *n* = 3 mice for sham group, *n* = 5 mice for PBS group, and *n* = 6 mice for pDA‐MNOF group, respectively. Data were presented with mean ± s.d.; ***, *p* < 0.001; ANOVA.

To investigate whether pDA‐MNOF was able of upregulating HO1 and SOD2 after being implemented in vivo, 12.5 µg mL^−1^ pDA‐MNOF was injected into lateral ventricles of MCAo mice, whose brain tissues around infarct were collected for further analysis. Compared to PBS treatment, the mRNA levels of HO1 and SOD2 exhibited the 1.87‐fold and 9.64‐fold increase in pDA‐MNOF group (**Figure** **5**a), respectively. We also examined the protein levels of p‐STAT3, HO1, and SOD2 in tissue lysate samples of the two groups. All of four sample in pDA‐MNOF group exhibited the higher protein levels of p‐STAT3, HO1, and SOD2 than those in PBS control (Figure 5b,c), demonstrating that pDA‐MNOF activates the two antioxidant enzymes and STAT3 signaling pathway in vivo.

**Figure 6:**
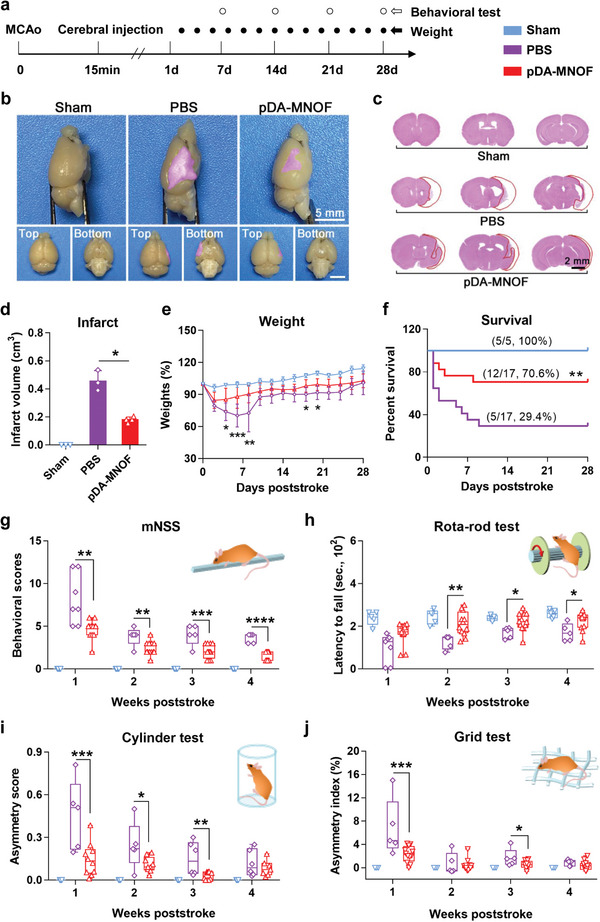
pDA‐MNOF reduces stroke cavity and promotes the poststroke behavioral recovery. a) The experimental flowchart shows MCAo operation, stereotactic injection, weighting, and behavioral tests. b) Appearance of murine brains with or without stroke cavity (purple) in indicated groups. Scale bars, 5 mm. c) Hematoxylin and eosin stained brain section of mice in indicated groups. Stroke cavity region in each brain section was outlined by red lines. Scale bar, 2 mm. d) Quantification of infarct volumes in (c). *n* = 3 mice for sham group, *n* = 3 mice for PBS group, and *n* = 4 mice for pDA‐MNOF group, respectively. Data were presented with mean ± s.d.; *, *p* < 0.05; ANOVA. e) Weight changes and f) survival curves of the mice in different groups. *n* = 5 mice for sham group, *n* = 17 mice for PBS group, *n* = 17 mice for pDA‐MNOF group, respectively. Data were presented with mean ± s.d.; *, *p* < 0.05; **, *p* < 0.01; ***, *p* < 0.001; ANOVA. g) The mNSS test of these mice. Data were presented with mean ± s.d.; **, *p* < 0.01; ***, *p* < 0.001; ****, *p* < 0.0001; ANOVA. h) Rota‐rod test, i) cylinder test, and j) grid test for motor ability and the dexterity of forelimbs and hindlimbs of mice in different groups over 4 weeks, respectively. Data were presented with mean ± s.d.; *, *p* < 0.05; **, *p* < 0.01; ***, *p* < 0.001; ANOVA.

Since pDA‐MNOF possessed the SOD‐like activity and the capability of upregulating HO1 and SOD2, we sought to determine whether these nanozymes‐based therapies could reduce the in vivo ROS poststroke. The ROS levels in the lysis of fresh brain tissues were probed by DCFH, whose intensity was positively and quantitatively correlated with ROS. The fluorescence intensity in MCAo mice received pDA‐MNOF treatment was decreased by 25.9% compared to that in PBS treatment group (Figure 5d), suggesting that pDA‐MNOF effectively reduces in vivo ROS. However, the in vivo ROS‐scavenging effect of pDA‐MNOF was weakened when being combined with AG490 (Figure 5d), indicating that pDA‐MNOF‐induced antioxidant enzyme activation through STAT3 signaling pathway is involved in ROS scavenging in vivo. Further, in these MCAo mice, the proportion of apoptotic cells in the pDA‐MNOF group stained by terminal deoxynucleotidyl transferase dUTP nick end labeling (TUNEL) assay was 29.9%, much lower than 64.6% of the PBS group (Figure 5e,f), demonstrating that pDA‐MNOF reduces neural cell death. Moreover, cerebral injection of pDA‐MNOF significantly reduced infarct volumes by 64.5% (165.2 mm^3^ vs 464.7 mm^3^, 2,3,5‐triphenyltetrazolium chloride (TTC) staining assay) (**Figure** **6**g,h and Figure S17a–c, Supporting Information), compared to PBS treatment. Collectively, these results demonstrate that pDA‐MNOF treatment effectively lowers ROS levels, neuron death, and poststroke infarct volume.

### 2.6 pDA‐MNOF Reduces Volumes of Stroke Cavity and Improves Functional Recovery

To test the long‐term therapeutic effect of pDA‐MNOF, a set of tests was performed on MCAo mice (Figure 6a). During the chronic phase of stroke, infarct brain tissues often form a cavitary lesion, whose size is closely associated with illness condition.^[^ ^40^ ^]^ 28 days after different treatments, compared to PBS control, the brain of mice in pDA‐MNOF group had significantly smaller lesion region (Figure 6b and Figure S18a–c, Supporting Information), with a decrease of 60.5% (180.1 mm^3^ vs 456.4 mm^3^) (Figure 6c,d and Figure S19a–c, Supporting Information). These results suggest that pDA‐MNOF treatment significantly lowers the formed cavitary region in chronic phase of stroke.

Given that mice typically suffer weights loss after stroke,^[^ ^41^ ^]^ we monitored body weight changes over a 4 week period. Compared to PBS control, MCAo mice receiving pDA‐MNOF treatment exhibited less weight loss and faster weight recovery (Figure 6e). All of the deaths occurred within the first 9 days of stroke, the overall survival ratio of pDA‐MNOF group was 70.6%, much higher than 29.4% of PBS control (Figure 6f), suggesting the excellent capability of these nanozymes on reducing mortality. The modified Neurological Severity Scores (mNSS), which comprehensively reflect performance of movement, balance, sensory, and reflex, were utilized to evaluate neurological defects. The lower scores indicate enhanced functional outcomes. Compared to PBS control, pDA‐MNOF‐treated mice had the lower mNSS at Week 1, 2, 3, and 4 (Figure 6g). In Rota‐rod test, the latency to fall in the mice of pDA‐MNOF group was much higher than that in PBS control at Week 2, 3, and 4 (Figure 6h), indicating the improved motor coordination. Additionally, the dexterity of contralateral forelimb and hindlimb of MCAo mice was evaluated in cylinder test and grid test. The MCAo mice receiving pDA‐MNOF treatment had the decreased asymmetry scores at Week 1, 2, and 3 (Figure 6i), and the reduced asymmetry index at Week 1 and 3 poststroke (Figure 6j), suggesting the improved dexterity of their limbs. Taken together, the four independent behavioral tests comprehensively demonstrate that pDA‐MNOF treatment significantly promotes the behavioral recovery of stroke mice.

### 2.7 pDA‐MNOF Enhances Vascular Endothelial Growth Factor (VEGF) Secretion and In Vivo Angiogenesis Poststroke

**Figure 7:**
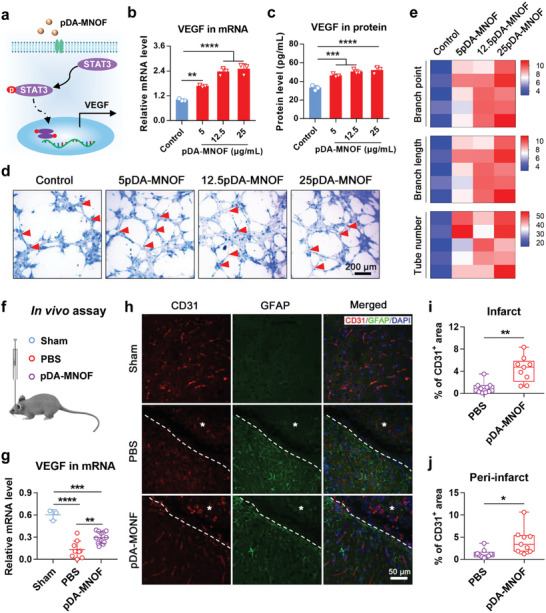
pDA‐MNOF promotes angiogenesis. a) Schematics showing VEGF, a downstream gene of STAT3 signaling, was activated by pDA‐MNOF. b) The mRNA levels of VEGF in N2a cells treated with PBS (control) or pDA‐MNOF (*n* = 3). Data were presented with mean ± s.d.; **, *p* < 0.01; ****, *p* < 0.0001; ANOVA. c) The levels of VEGF protein secreted from the PBS (control) or pDA‐MNOF‐treated N2a cells (*n* = 3). Data were presented with mean ± s.d.; ***, *p* < 0.001; ****, *p* < 0.0001; ANOVA. d) Capillary‐like tube formation assay performed on C166 cells (murine endothelial cell line) that were cultured with regular N2a culture medium or conditioned medium obtained from the pDA‐MNOF‐treated N2a cells. 5pDA‐MNOF, 12.5pDA‐MNOF, or 25pDA‐MNOF represent conditioned medium that was from the 5, 12.5, or 25 µg mL^−1^ pDA‐MNOF‐treated N2a cells, respectively. The branch points were indicated by red arrowheads, and the structure between two adjacent arrowheads was a branch. e) Quantification of branch point numbers, tube length, and tube numbers in capillary‐like tube formation assay in (d) (*n* = 5 repeats). f) Schematics of cerebral ventricle injection of PBS and pDA‐MNOF for further evaluating in vivo angiogenesis. g) Relative VEGF mRNA levels in brain tissue of the MCAo mice receiving cerebral ventricle injection of PBS or pDA‐MNOF (*n* = 9). The mice received sham operation were set as control (*n* = 3). Data were presented with mean ± s.d.; **, *p* < 0.01; ***, *p* < 0.001; ****, *p* < 0.0001; ANOVA. h) Representative fluorescence images of vessels (CD31, red) and astrocytes (GFAP, green) in the infarct (an asterisk) and peri‐infarct 4 weeks after the mice received cerebral ventricle injection of PBS or pDA‐MNOF. The boundary between the infarct and peri‐infarct was indicated by white dashed lines. i,j) Quantification of percentages of CD31 positive (CD31^+^) area in i) the infarct and j) peri‐infarct, respectively (*n* = 9). Data were presented with mean ± s.d.; *, *p* < 0.05; **, *p* < 0.01; ANOVA.

STAT3 signaling, which was activated in pDA‐MNOF‐treated neuronal cells, is closely associated with angiogenesis through promoting VEGF transcription^[^ ^37^ ^]^ (**Figure** **7**a). Thus, to determine whether pDA‐MNOF enhanced angiogenesis, we examined the mRNA and protein levels of VEGF in pDA‐MNOF‐treated N2a cells, and assessed activities of the secreted VEGF through a capillary‐like tube formation assay. Elevated mRNA levels of VEGF were observed in N2a cells treated with 12.5 and 25 µg mL^−1^ pDA‐MNOF (Figure 7b), indicating that pDA‐MNOF activates VEGF transcription. We collected the culture medium of N2a cells in these groups and tested the protein levels of VEGF using a commercial enzyme‐linked immunosorbent assay (ELISA) kit. Compared with PBS control, all of the three pDA‐MNOF‐treated groups showed higher VEGF concentration in culture medium (Figure 7c). No obvious capillary‐like tubes were formed in C166 cells (murine endothelial cells) that were treated with N2a cells’ culturing medium. In contrast, extensive and enhanced capillary‐like tube formation were found in the three pDA‐MNOF groups, as evidenced by the increase of branch points, total tube length, and tube numbers (Figure 7d,e).

Next, to investigate whether pDA‐MNOF promoted VEGF expression and angiogenesis after implementation in vivo, the mRNA detection and CD31 (a marker of vascular endothelial cells) staining were performed (Figure 7f). Consistent with results of in vitro assay, pDA‐MNOF treatments significantly enhanced VEGF expression in mRNA levels (Figure 7g). For CD31 staining test, the increased proportion of CD31 stained area was observed in the pDA‐MNOF group either in the infarct or in the peri‐infarct (Figure 7h–j), suggesting that pDA‐MNOF treatment promoted in vivo angiogenesis poststroke.

### 2.8 In Vivo Biocompatibility of pDA‐MNOF

**Figure 8:**
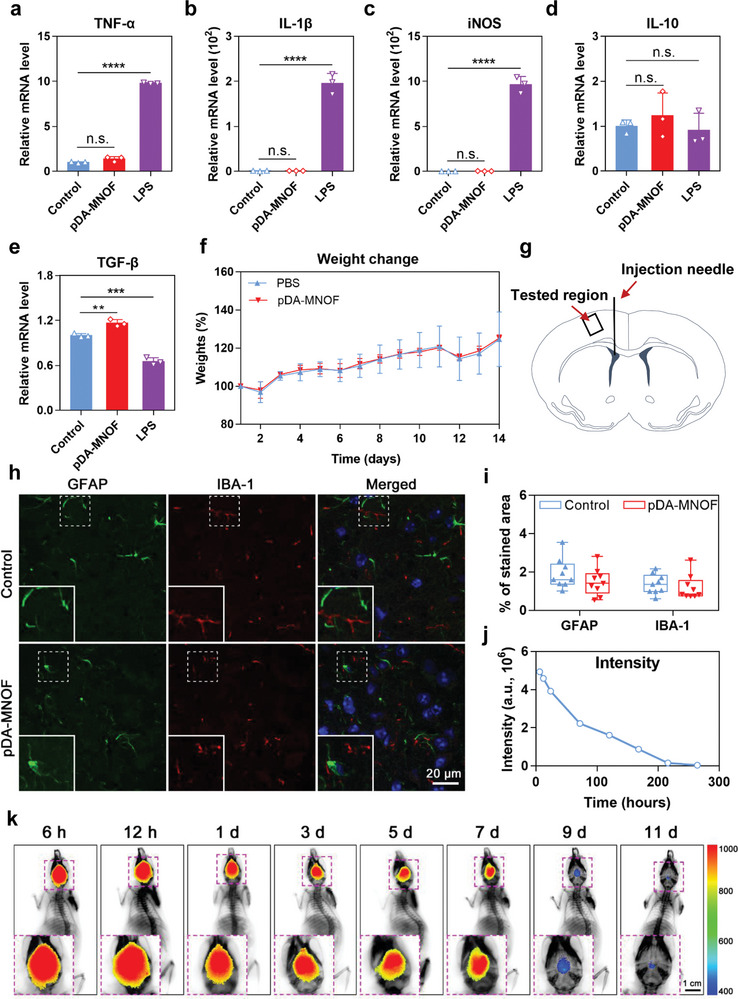
In vivo biocompatibility of pDA‐MNOF. a–e) The mRNA levels of a) TNF‐*α*, b) IL‐1*β*, c) iNOS, d) IL‐10, and e) TGF‐*β* in BV‐2 cells 24 h after being treated with PBS (control), pDA‐MNOF (12.5 µg mL^−1^), or LPS (2 µg mL^−1^) (*n* = 3 repeats). Data were presented with mean ± s.d.; n.s., not significant; **, *p* < 0.01; ***, *p* < 0.001; ****, *p* < 0.0001; ANOVA. f) Weights of the mice receiving cerebral injection of PBS or pDA‐MNOF at different time points. *n* = 5 mice for PBS group, and *n* = 6 mice for pDA‐MNOF group. g) Schematics showing detection of activation of astrocytes and microglia in the brain of the mice receiving pDA‐MNOF injection. The tested region was boxed with black lines, and the injection needle was indicated by a black line. h) Immunofluorescence staining of brain tissues in the tested region (g) for GFAP (astrocytes, green), IBA‐1 (microglia, red), and DAPI (nuclei, blue) in the different groups. Regions boxed with white dashed lines were enlarged and placed on lower left corners. Scale bar, 20 µm. i) Quantification of proportions of GFAP positive area and IBA‐1 positive area in (h) (*n* = 9 sections). j) Quantification of fluorescent intensity of ICG@pDA‐MNOF (excitation/emission wavelength, 760 nm/830 nm) in the brains at different time points after intracerebroventricular injection. k) Representative fluorescence images of the mice after receiving cerebral injection of ICG@pDA‐MNOF for different time durations. The brain regions boxed by purple dotted lines were enlarged and placed on the lower left corner. Scale bar, 1 cm. The color bar representing the fluorescent intensity values corresponding to different colors was placed on the right.

To investigate whether pDA‐MNOF could cause inflammatory reaction, the mRNA expression of inflammatory‐associated cytokines in microglia, a main type of inflammatory cells in brain, was tested. Three pro‐inflammatory cytokines, including TNF‐*α*, IL‐1*β*, and iNOS, were differentially elevated in lipoploysaccharide (LPS)‐treated BV‐2 cells, but remained at low levels in pDA‐MNOF‐treated cells (**Figure** **8**a–c). We also tested the mRNA expression of IL‐10 and TGF‐*β*, two vital anti‐inflammatory cytokines, for comprehensively characterizing the impact of pDA‐MNOF on inflammatory reaction. There was no significant difference in IL‐10 levels between the pDA‐MNOF group and the PBS control, but slightly elevated mRNA expression of TGF‐*β* was observed in these pDA‐MNOF‐treated BV‐2 cells (Figure 8d,e). These results demonstrate that pDA‐MNOF treatment appears to slightly suppress the microglia‐mediated inflammatory reaction. The similar results were found in the pDA‐MNOF‐treated brain tissues (Figure S20, Supporting Information). Given that hyper‐inflammatory responses often occur and lead to neurons death and brain edema following stroke, pDA‐MNOF with mild inflammation‐suppressing activity might benefit stroke treatment.

To further determine the effect of pDA‐MNOF on in vivo inflammatory reaction, the mice were subjected to receive cerebral injection of these nanozymes. The pDA‐MNOF‐injected mice did not exhibit a noticeable weight decline (Figure 8f), indicating that this nanomaterial has no obvious side effects after being delivered in vivo. As two main types of cells that were positively responsive to inflammatory reaction,^[^ ^42^, ^43^, ^44^ ^]^ astrocytes and microglia in ipsilateral cortex were analyzed (Figure 8g). There was no difference in GFAP (a marker of astrocytes) and IBA‐1 (a marker of microglia) stained areas between the mice receiving pDA‐MNOF and those receiving PBS (Figure 8h,i), suggesting no obvious activation of astrocytes and microglia. Moreover, the biosafety of pDA‐MNOF treatment was verified as evidenced by the results of cell density of neurons in tested regions (Figure S21a,b, Supporting Information).

To visualize and track the pDA‐MNOF after being cerebral injected in vivo, we modified the nanozymes with indocyanine green (ICG), a type of near‐infrared fluorescent dye, during their synthesis process. The fluorescent intensity of ICG@pDA‐MNOF gradually decreased after 3 days and nearly disappeared at Day 9 (Figure 8j,k), suggesting that pDA‐MNOF can be cleared from the brain. Additionally, the results of inductively coupled plasma mass spectrometry (ICP‐MS) revealed the gradual decrease of manganese over time (Figure S22, Supporting Information), further confirming its in vivo clearance. The in vivo clearance of pDA‐MNOF supports the bio‐safety of this nanozyme‐based therapy.

## 3 Conclusions

In summary, we developed an MOF‐based nanozyme with SOD‐like catalytic activities capable of upregulating SOD2 and HO1 through activating STAT3 signaling. When applied to the preclinical stroke model, MCAo, pDA‐MNOF‐based therapy could significantly improve animal survival, reduce infarct volumes, and achieve enhanced behavioral outcomes. In addition, the angiogenesis‐promoting effects of pDA‐MNOF were uncovered, which favored brain tissue repair. Such a novel nanozyme‐based therapy with potent ROS‐scavenging activities might be a valuable alternative for stroke treatment.

## 4 Experimental Section

### Chemicals and Reagents

Ethanol (EtOH), manganese chloride tetrahydrate (MnCl_2_·4H_2_O), cupper (II) chloride dihydrate (CuCl_2_·2H_2_O), concentrated aqueous ammonia (NH_4_OH), nickel chloride hexahydrate (NiCl_2_·6H_2_O), triethylamine, dopamine, acetone, *tris*(hydroxymethyl)methyl aminomethane (Tris) were purchased from China National Medicines Corporation LTD., which were used without further purifications. 2,3,6,7,10,11‐Hexaiminotriphenylene hexahydrochloride (HATP·6HCl) was purchased from Jilin Chinese Academy of Sciences‐Yanshen Technology Co., Ltd.

### Synthesis of MNOF, Cu‐MNOF, and pDA‐MNOF

A mixture of 11.8 mg (0.06 mmol) of MnCl_2_·4H_2_O, 20.0 mg (0.040 mmol) of HATP·6HCl, and 2 mL of triethylamine dissolved in 30 mL deionized water. Then, the mixture was stirred for 2 days at 70 °C in an open flask. The resulting product was centrifuged, and then washed in water under reflux for 24 h. Then, the product was heated at reflux in acetone for another 2 h. Finally, the MNOF was obtained after being dried under vacuum at 60 °C. To obtain Cu‐MNOF, 20 mg dried MNOF was added in 100 mL deionized water containing 0.02 mg mL^−1^ CuCl_2_. Then, the mixture was stirred at room temperature overnight. After being centrifuged, filtered, and washed, the solid was collected and then heated at reflux in acetone for 3 h. Finally, the product was dried in vacuum at 60 °C to yield Cu‐MNOF. For the synthesis of the pDA‐MNOF, 50 mg dopamine was added to 80 mL tris‐buffer solution, which was stirred vigorously for 1 h at room temperature. Then the 400 mg MNOF was dispersed in the mixture and stirred for 48 h. The suspension was centrifugated and washed by deionized water and ethanol three times. Then, the sample was dried in vacuum at 60 °C overnight to yield pDA‐MNOF.

### Characterization of These Nanomaterials

The PXRD patterns were obtained on a Rigaku Smartlab using Cu K*α* radiation (45 kV, 40 mA). The scan speed was 3° min^−1^ at room temperature. TEM images of samples were acquired using a JEM‐1400 electron microscope. The IR analysis was performed using a Nicolet NEXUS670 IR spectroscopy. Scanning electron microscopy images of samples were obtained using a Helios G4 UC electron microscope. The sample was degassed under vacuum at 100 °C for 24 h prior to analysis, and then N_2_ sorption isotherm was collected using a surface area and pore size analyzer (Quantachrome Autosorb‐1). TGA was performed on a TA Instruments Q‐500 series thermal gravimetric analyzer, and samples were heated at a constant rate of 12 °C min^−1^ during TGA test. DLS was carried out with a ZEN3700 system. The quantitative analysis of Cu^2+^ content in Cu‐MNOF was done using an Agilent 700 inductively coupled plasma optical emission spectrometer. Elemental analysis of samples was performed using a Perkin‐Elmer 2400 II CHNS device. DFT chemical calculations, including geometry optimizations and frequency calculations, were performed at the B3LYP/6‐31G* level with the D3 version of Grimme's dispersion with Becke‐Johnson damping in the quantum chemical package Gaussian 09.

### Measurement of the SOD‐Like Activity

Commercial superoxide dismutase detection kit (A001‐3, JianCheng, Nanjing) was used to test SOD‐like activities of these MOFs. Briefly, WST‐1 was used as an indicator to detect the amount of O_2_^●−^, which were produced by X and XO. Before the reaction, different concentrations of MONF or pDA‐MNOF were added into the mixture and incubated at 37 °C for 20 min. After analysis of the absorbance of supernatant at 450 nm, the SOD‐like activity of MOFs was calculated. In addition, NBT, another probe for testing superoxide ions, was used to evaluate the O_2_^●−^ scavenging activities of MOFs. Different concentrations of MNOF or pDA‐MNOF and NBT (0.075 × 10^−3^ m) were mixed with riboflavin (0.02 × 10^−3^ m) and methionine (13 × 10^−3^ m) in PBS buffer (0.25 × 10^−3^ m). Then, the mixture was irradiated with a sunlamp for 2 min, and the absorbance at 550 nm was recorded. In addition, the ESR assay was performed to verify the O_2_^●−^ scavenging activities of MNOF. Briefly, 5‐diethoxyphosphoryl‐5‐methyl‐1‐pyrroline N‐oxide (DEPMPO) was used as the O_2_^●−^ trapper. Then, the KO_2_ solution was added into an ethanol solution containing ethylenediaminetetraacetic acid (0.2 × 10^−3^ m), DEPMPO (40 × 10^−3^ m), and MNOF (2.5 or 5 µg mL^−1^), and the EPR spectra were recorded.

### Measurement of HO1‐Like, CAT‐Like, and GPX‐Like Activities of MNOF or pDA‐MNOF

To test the HO1‐like activities of these nanozymes, 1.25 µg of MNOF or pDA‐MNOF was added into 100 µL of 100 × 10^−3^ m Tris‐HCl containing 15 × 10^−6^ m hemin, 0.8 × 10^−3^ m NADPH, 1 × 10^−3^ m MgCl_2_, 0.8 × 10^−3^ m glucose 6‐phosphate, 300 × 10^−6^ m bovine serum albumin (BSA), 1 U glucose‐6‐phosphate dehydrogenase, and 5 × 10^−6^ m biliverdin reductase for 30 min. Then, the fluorescence intensity was recorded with the excitation and emission wavelengths of 441 and 528 nm. For CAT‐like activities test, 12.5 µg nanozymes were added into 1 mL PBS containing 100 × 10^−3^ m H_2_O_2_ for a 20 min incubation. After centrifugation at 12 000 rpm for 10 min, the supernatants were obtained. Then, the remaining H_2_O_2_ was tested according to the instruction of Hydrogen Peroxide Assay Kit (S0038, Beyotime, China). To test the GPX‐like activities of these nanozymes, 5 µL MNOF or pDA‐MNOF was added into 178 µL of working solution containing 5 µL GSH and 12 µL Cum‐OOH for a 12 min incubation. Then, this mixture was reacted with 6.6 µL 5,5'‐dithio‐bis‐(2‐nitrobenzoic acid) solution for 10 min, and its absorbance was recorded at 412 nm.

### Measurement of the Scavenging Activities against Other Species of ROS

To test the ^●^OH scavenging activities, 12.5 µg mL^−1^ MNOF or pDA‐MNOF was added into 140 µL acetate buffer (pH 4.5) containing 20 µL FeSO_4_, 20 µL 3,3´,5,5´‐tetramethylbenzidine, and 20 µL 100 × 10^−3^ m H_2_O_2_ for a 10 min incubation, and the absorbance was recorded at 620 nm to reflect the remaining ^●^OH. Commercial singlet oxygen sensor green (SOSG) fluorescent probes were used to test the remaining ^1^O_2_. Briefly, 2.5 µg of MNOF or pDA‐MNOF was added into 200 µL distilled water containing 6 µL of SOSG and 6.4 µL of tri(chloro‐propyl) phosphate (250 µg mL^−1^). After being irradiated with sunlamp for 15 min, the fluorescence intensity was recorded with the excitation and emission wavelengths of 503 and 525 nm. For measurement of ^●^NO scavenging activity, the commercial nitric oxide detection kit (S0021S, Beyotime, China) was used. Briefly, 5 µg MNOF or pDA‐MNOF was added into the working solution containing 300 µL PBS (0.01 m, pH 7.4) and 100 µL sodium nitroprusside (SNP) for 1 h incubation. Then, 50 µL of Griess Reagent 1 and Reagent 2 were mixed with the equivalent volumes of the above solutions for 10 min, and the absorbance was recorded at 540 nm. To test the DPPH^●^ scavenging activities, 5 µg of MNOF or pDA‐MNOF was added into the 400 µL acetate buffer (100 × 10^−3^ m, pH 5.5) containing 0.15 × 10^−3^ m DPPH. After incubation for 40 min in the dark, the absorbance was recorded at 517 nm.

### Cell Culture and Treatment

Murine neuroblastoma cells (N2a), microglial cells (BV‐2), and vascular endothelial cells (C166) were cultured in DMEM (Hyclone, USA) supplemented with 10% FBS (Gibco, USA) at a humidified incubator with 5% CO_2_. To inhibit STAT3 signaling, N2a cells were pretreated with 10 × 10^−6^ m AG490 (MedChemExpress, USA) for 12 h and then incubated with pDA‐MNOF for following 12 h for further analyses. Moreover, RNA interfering technique was utilized to silence STAT3, HO1, and SOD2. Briefly, N2a cells were seeded at the density of 2.5 × 10^5^ cells per well of a 12‐well plate and then transfected with the 100 × 10^−9^ m specific siRNA or negative control siRNA (Ribo, China) using Lipofectamine 3000 (Thermo Fisher Scientific, USA) for a 60 h incubation. Then, the cells were treated with pDA‐MNOF for 12 h and collected for further analyses.

### qRT‐PCR, Western Blot (WB) Assay, and Immunofluorescence Staining

For quantitative reverse transcription polymerase chain reaction (qRT‐PCR) assay, the pDA‐MNOF‐treated N2a cells or the LPS‐treated BV‐2 cells were collected for extracting the total RNA with Trizol reagent (Invitrogen, USA). 3 µg of mRNA was reversely transcribed to cDNA, and the following qRT‐PCR test (Vazyme, China) was performed to evaluate the mRNA expression of specific genes. The primer sequences were displayed in Table S1 in the Supporting Information. For WB assay, cells or tissues were treated with radioimmunoprecipitation assay buffer (Sigma‐Aldrich, Germany) containing the inhibitors of proteinase and phosphatase to obtain the lysed samples. The protein concentrations of lysates were determined using the BCA protein assay reagent (Pierce, USA). 30 µg protein of each sample was separated with sodium dodecyl sulfate‐polyacrylamide gel electrophoresis (12%, w/v) and then transferred onto the nitrocellulose membranes (Amersham, USA). The membranes were blocked with 5% BSA (Biosharp, China) for 1 h, and incubated overnight with the following antibodies: anti‐*β*‐actin (1:5000 diluted, 10230‐1‐AP, Proteintech, China), anti‐p‐STAT3 (1:3000 diluted, Tyr705; AF3293, Affinity Biosciences, USA), anti‐STAT3 (1:3000 diluted, 60199‐1‐Ig, Proteintech, China), anti‐HO1 (1:3000 diluted, 66743‐1‐Ig, Proteintech, China), anti‐SOD2 (1:1000 diluted, 66474‐1‐Ig, Proteintech, China). After washing three times with TBST, the membranes were incubated with anti‐mouse secondary antibodies (1:5000 diluted, Proteintech, China) or anti‐rabbit secondary antibodies (1:5000 diluted, Proteintech, China) for 2 h at room temperature. After washing three times, blots were analyzed using a BioSpectrum600 Imaging System (Upland, USA). Immunofluorescence staining was performed on pDA‐MNOF‐treated N2a cells for detecting expression of p‐STAT3, HO1, and SOD2. These treated N2a cells were fixed with 4% paraformaldehyde for 15 min at room temperature. After being washed with PBS for three times, cells were incubated for 10 min with PBS containing 0.1% Triton X‐100 for permeabilization. Then, cells were incubated with 5% BSA for 30 min to block unspecific binding of antibodies. After being washed with PBS for three times, cells were incubated with indicated primary antibodies overnight at 4 °C and then incubated with anti‐mouse or anti‐rabbit secondary antibodies (1:500 diluted, Proteintech, China) at room temperature for 1 h. The nuclei were stained with 4′,6‐diamidino‐2‐phenylindole (DAPI, Boster, China) for 20 min. All the samples were observed using a confocal laser scanning microscope (A1Si, Nikon, Japan).

### Establishment of an OGD Model and In Vitro Cell Assay

The in vitro OGD model was constructed as previously described.^[^ ^45^ ^]^ Briefly, the culture plate was placed in a sealed bag containing an anaerobic bag (GENbag anaer, bioMerieux, France) that continuously consumed oxygen, and N2a cells were incubated in glucose‐free DMEM (Gibco, USA) without FBS on these culture plates for 8 h. Then the cells were cultured in DMEM containing 10% FBS at regular conditions for 16 h. Thus, the in vitro OGD model was established. Under the OGD, N2a cells were treated with PBS or various concentrations of pDA‐MNOF. Live & dead staining (Biovision, USA) and CCK‐8 assay were performed to evaluate cell viability of N2a cells. For CCK‐8 assay, 100 µL of CCK‐8 working solution was added into each well for a 30 min incubation. To eliminate the effect of nanomaterials on this test, 50 µL of supernatant was carefully transferred from the upper layer of medium in each well to a new 96‐well plate. The absorbance was recorded at 450 nm. To test intracellular ROS levels, N2a cells suffered an 8 h anaerobic incubation were treated with DCFH‐DA (JianCheng, Nanjing) for 20 min, and then washed with PBS three times. These stained cells were analyzed using an inverted fluorescence microscope (IX 71, Olympus, Japan) and an FACS Calibur flow cytometer (BD Canto II, USA), and the fluorescence intensity was recorded with the excitation and emission wavelengths of 492 and 515 nm, respectively.

### In Vivo Treatment of MCAo Mice

All animal experiments were performed in strict accordance with the guidelines for the care and use of laboratory animals (ethics committee of Tongji Medical College, Huazhong University of Science and Technology) and approved by the animal care and use committee (2022, IACUC Number: 2835) of Huazhong University of Science and Technology, Wuhan, China. The C57BL/6 mice were anesthetized and placed on a heating pad to keep their body temperature. The head of mice were fixed with a stereotaxic apparatus (RWD, China), and a hole was drilled into skull along the anterior‐posterior axis at the coordinates (mm): anterior‐posterior, −2.0; medial‐lateral, +2.5; dorsal‐ventral, −1.0. After the MCAo was induced by a silicone‐coated filament, the mice received the cerebral ventricle injection of 5 µL pDA‐MNOF solution through a Hamilton syringe with a 33‐G needle. Cerebral artery was blocked for 60 min in these MCAo mice. Then the embolus was pulled out and the skin incision was sutured. All of mice were kept on a heating pad until they recovered from anesthesia. The weight and survival of these MCAo mice were observed and recorded for 4 weeks.

### ROS Detection, TTC, and TUNEL Staining

OxiSelect in vitro ROS/RNS assay kit (STA‐347, Cell Biolabs, USA) was used to detect the ROS in ischemic brain tissues. 3 mg of ischemic brain tissues of MCAo mice receiving different treatments were collected 10 min after reperfusion. After being homogenized in 200 µL ice‐cold PBS, 50 µL of catalyst solution was added into the mixture to accelerate the oxidation reaction. Then, 100 µL of DCFH‐DiOxyQ probe solution was added and incubated at room temperature for 30 min, and the fluorescence intensity was recorded with the excitation and emission wavelengths of 492 and 515 nm.

The infarct volume of brains in these MCAo mice was examined through TTC staining. Mice were sacrificed at 24 h following MCAo operation, and the intact brains were gently taken out, frozen at −20 °C for 30 min, and cut into 2 mm thick coronal slices. Then, these brain slices were immersed into 1% TTC solution (Sigma, USA) at 37 °C and turned over after a 15 min incubation. These stained brain slices were imaged using a scanner (LIDE300, Canon, Japan). TUNEL staining (in situ cell death detection kit, Roche, Swiss) was performed according to the manufacturer's protocols. The nuclei were stained with DAPI for 20 min. All the samples were observed using an inverted fluorescence microscope.

### Assessment of Behavioral Performance

All of these MCAo mice were examined using these four behavioral tests at Week 1, 2, 3, and 4. The mNSS were determined according to a 14‐point rating scale as previously described,^[^ ^46^ ^]^ with lower scores indicating better functional outcomes. In the Rota‐rod test,^[^ ^47^ ^]^ the mice were pretrained on a Rota‐rod with an accelerating speed from 4 rpm to 40 rpm before MCAo surgery for 1 week. During the test, the MCAo mice were placed on this Rota‐rod with the same parameter, and the latency to fall within 5 min was recorded. In the cylinder test,^[^ ^48^ ^]^ the MCAo mice were in a 15 cm high transparent cylinder with a diameter of 10 cm, and the vertical exploratory movement of mice was videotaped for 5 min by a camera. The duration (seconds) of each forelimb placed on the wall was recorded for the following analysis. The difference between the ipsilateral use time and contralateral use time was divided by the total use time of the forelimb, and therefore an asymmetry score was obtained. In the grid test,^[^ ^49^ ^]^ MCAo mice were placed on a stainless steel grid (32 cm × 20 cm) and allowed to walk freely for 5 min, and each movement was recorded by a camera under the grid. During the walk, one step when the foot went through the grid hole was defined as a footfault. The difference between the contralateral footfaults and the ipsilateral footfaults was divided by total steps, and therefore an asymmetry index was acquired.

### In Vitro and In Vivo Evaluation of Angiogenesis

N2a cells were treated with PBS or a series of pDA‐MNOF (5, 12.5, or 25 µg mL^−1^) for 12 h, then total mRNA was extracted and the mRNA levels of VEGF in indicated groups were tested through qRT‐PCR assay. To assess the protein levels of VEGF, the supernatant of N2a cells that were treated with PBS or various concentrations of pDA‐MNOF for 24 h was collected for ELISA detection (Elabscience, China). Capillary‐like tube formation assay was performed to investigate the biological activity of VEGF induced by pDA‐MNOF. Briefly, N2a cells were treated with PBS or pDA‐MNOF for 24 h and the supernatant was collected. 200 µL of Matrigel (BD biosciences, USA) was added into each well of a 48‐well plate, and C166 cells combined with the collected supernatant medium were added into these wells for a 4 h incubation at 37 °C. After being stained with 0.5% crystal violet, C166 cells were observed with an inverted microscope and the related index was calculated with the help of ImageJ software (1.48v, NIH, USA). To evaluate the in vivo angiogenesis‐promoting effect of pDA‐MNOF, the brain tissues of MCAo mice 4 weeks after receiving pDA‐MNOF treatment were collected, and immunofluorescence staining of CD31 (1:500 diluted, Ab182981, Abcam, UK) and GFAP (1:500 diluted, GB12096, Servicebio, China) for the paraffin‐embedded brain slice was performed. All the stained brain slices were observed using a confocal laser scanning microscope.

### In Vitro and In Vivo Biocompatibility

To test whether pDA‐MNOF caused inflammatory responses, BV‐2 cells were treated with PBS, pDA‐MNOF (12.5 µg mL^−1^), or LPS (2.0 µg mL^−1^) for 24 h. The mRNA levels of TNF‐*α*, IL‐1*β*, iNOS, IL‐10, TGF‐*β* of N2a cells in indicated groups were examined through qRT‐PCR assay as previously described.^[^ ^45^ ^]^ Then, in vivo biocompatibility of pDA‐MNOF was tested. After anesthetization, the heads of C57BL/6 mice were fixed with a stereotaxic apparatus, and 0.5 µL of PBS or pDA‐MNOF (12.5 µg mL^−1^) was stereotactically injected into cerebral ventricle with a Hamilton syringe. Body weight was recorded daily through 2 weeks. Finally, the mice were anesthetized, perfused with PBS, and fixed with 4% paraformaldehyde (w/v, PBS). The fixed brains were embedded in paraffin and then cut into 4 µm thick slices. Immunofluorescence staining of GFAP (GB12096, Servicebio, China), NeuN (Ab 177487, Abcam, UK), and IBA‐1 (Ab178846, Abcam, UK) for the brain slice was performed according to previously described methods.^[^ ^45^ ^]^

### In Vivo Clearance of pDA‐MNOF

To visualize and track pDA‐MNOF after being implanted in vivo, these nanomaterials were modified with ICG, a type of near‐infrared fluorescent dye. Briefly, 10 mg of pDA‐MNOF and 2 mL of ICG solution were added into 20 mL distilled water, and then the mixture was stirred at room temperature overnight. After being centrifuged and washed with distilled water, the ICG@pDA‐MNOF was obtained. C57BL/6 mice were anesthetized and received the cerebral ventricle injection of 5 µL of ICG@pDA‐MNOF. At different time points, these treated mice were examined using a versatile small animal imaging system (In‐Vivo FX PRO, Bruker, Germany). The in vivo fluorescent signal of pDA‐MNOF was analyzed with the Bruker MI SE software. To test the dynamic changes of manganese in brains of these mice, the ICP‐MS was performed. Briefly, the brains of MCAo mice received the cerebral ventricle injection of 5 µL of pDA‐MNOF were collected at different time points, then frozen at −20 °C and sent to Shiyanjia Lab (www.shiyanjia.com) for the ICP‐MS measurement.

### Statistical Analysis

Data were presented as mean ± s.d., and one‐way analysis of variance (ANOVA) for multiple comparison or a two‐tailed Student's T‐test for comparison of two groups was used to assess the differences between experimental groups. Statistical analysis was performed using Graphpad Prism software (version 9.4.1.). The threshold for statistical significance was set to *p* < 0.05.

## Conflict of Interest

The authors declare no conflict of interest.

## Supporting information

Supporting Information

## Data Availability

The data that support the findings of this study are available from the corresponding author upon reasonable request.
