# Supplementary material for: A Bioinspired Manganese‐Organic Framework Ameliorates Ischemic Stroke through its Intrinsic Nanozyme Activity and Upregulating Endogenous Antioxidant Enzymes
Source: Adv Sci (Weinh). 2023 May 2;10(20):2206854. doi: 10.1002/advs.202206854 (PMC10369237; doi:10.1002/advs.202206854)
Supplement: Supplementary file 1 — Supporting Information [file ADVS-10-2206854-s001.pdf]

ADVANCED SCIENCE Open Access

Supporting Information

for *Adv. Sci.*, DOI 10.1002/advs.202206854

A Bioinspired Manganese-Organic Framework Ameliorates Ischemic Stroke through its Intrinsic Nanozyme Activity and Upregulating Endogenous Antioxidant Enzymes

Jian Wang, Yang Wang, Xiakeerzhati Xiaohalati, Qiangfei Su, Jingwei Liu, Bo Cai, Wen Yang, Zheng Wang and Lin Wang**

Supporting Information

A Bioinspired Manganese-Organic Framework Ameliorates Ischemic Stroke through Its Intrinsic Nanozyme Activity and Upregulating Endogenous Antioxidant Enzymes

Jian Wang, Yang Wang, Xiakeerzhati Xiaohalati, Qiangfei Su, Jingwei Liu, Bo Cai, Wen Yang, Zheng Wang, Lin Wang**

Prof. L. Wang, J. Wang, Q. Su
Department of Clinical Laboratory
Union Hospital
Tongji Medical College
Huazhong University of Science and Technology
Wuhan 430022, China

Prof. L. Wang, Prof. Z. Wang, J. Wang, Y. Wang, X. Xiaohalati, Q. Su, J. Liu, B. Cai, W. Yang,
Hubei Key Laboratory of Regenerative Medicine and Multi-disciplinary Translational Research
Research Center for Tissue Engineering and Regenerative Medicine
Union Hospital
Tongji Medical College
Huazhong University of Science and Technology
Wuhan 430022, China

Prof. Z. Wang
Department of Gastrointestinal Surgery
Union Hospital
Tongji Medical College

Huazhong University of Science and Technology

Wuhan 430022, China

E-mail: lin_wang@hust.edu.cn; zhengwang@hust.edu.cn

Keywords: MOFs, bioinspired nanozyme, antioxidant enzyme, ROS, stroke treatment

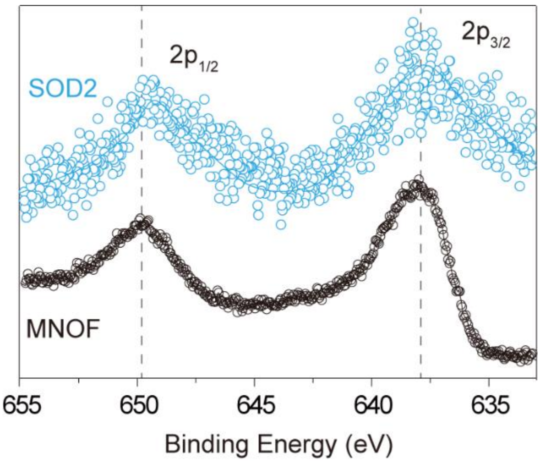

Figure S1. XPS spectra analysis of binding energy of manganese in SOD2 and MNOF.

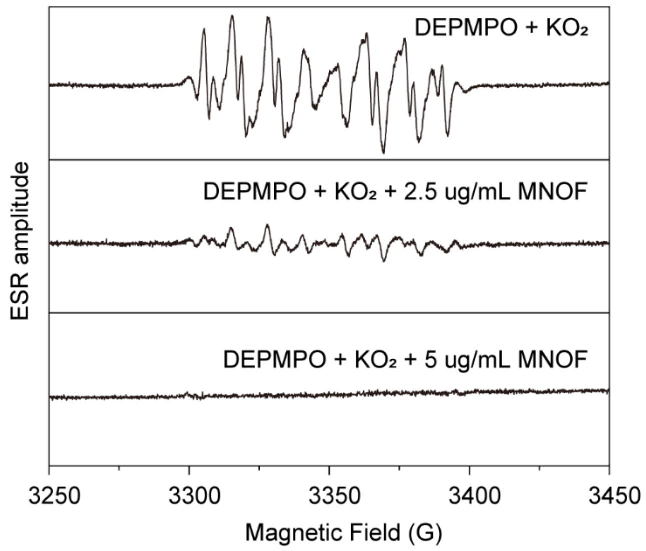

Figure S2. ESR spectra of the $O_2^{\bullet -}$ generation system containing 2.5 or 5 µg/mL MNOF.

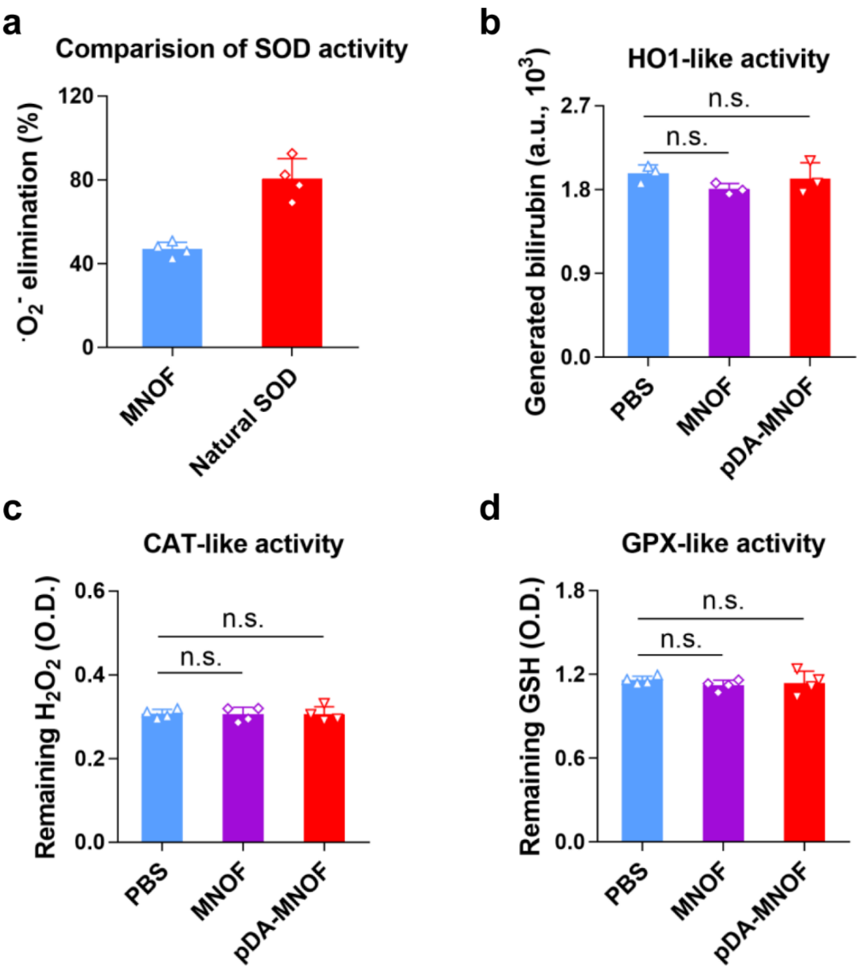

Figure S3. Enzyme-like activity detection. (a) Comparison of SOD activities between MNOF and natural SOD (n = 4). (b-d) Detection of the HO1-like (b, n = 3), CAT-like (c, n = 4) and GPX-like activities (d, n = 4) of MNOF and pDA-MNOF, respectively. Data were presented with mean ± s.d.; n.s., not significant; ANOVA.

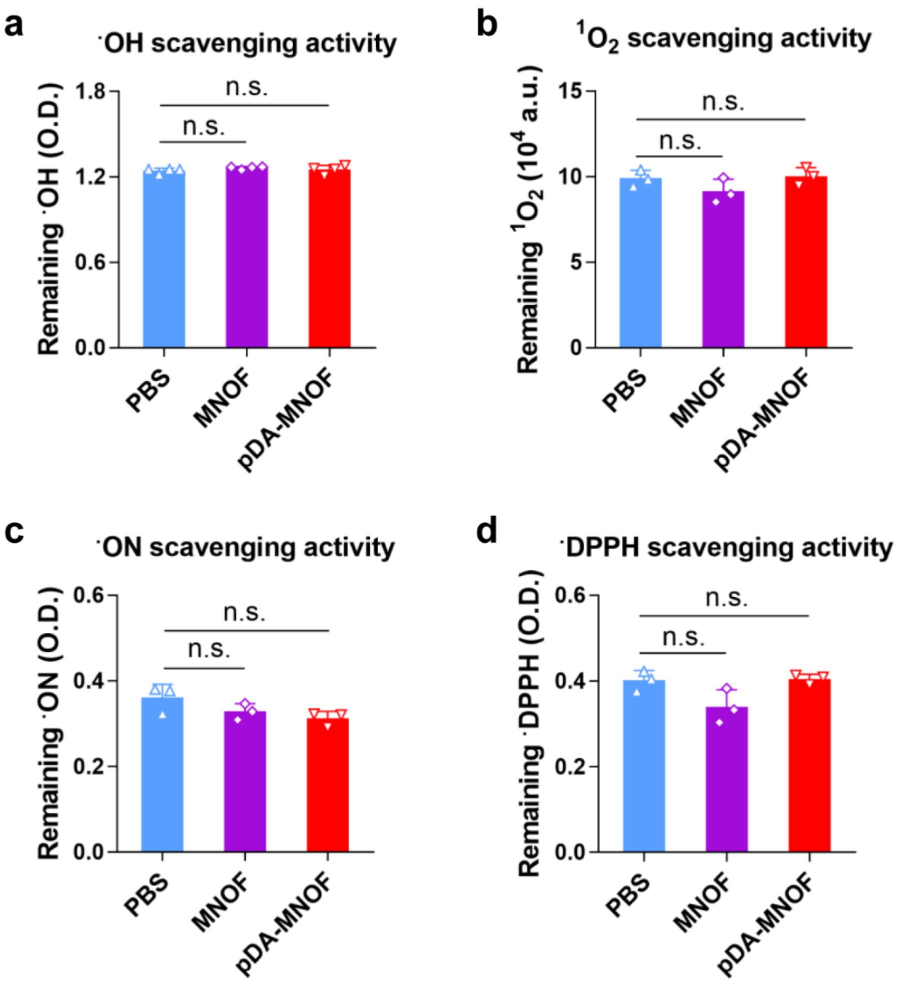

Figure S4. Measurement of ROS scavenging activities. (a-d) The scavenging activities of MNOF or pDA-MNOF against hydroxyl radical (a, n = 4), singlet oxygen (b, n = 3), nitroxide radical (c, n = 3), and DPPH radical (d, n = 3), respectively. Data were presented with mean ± s.d.; n.s., not significant; ANOVA.

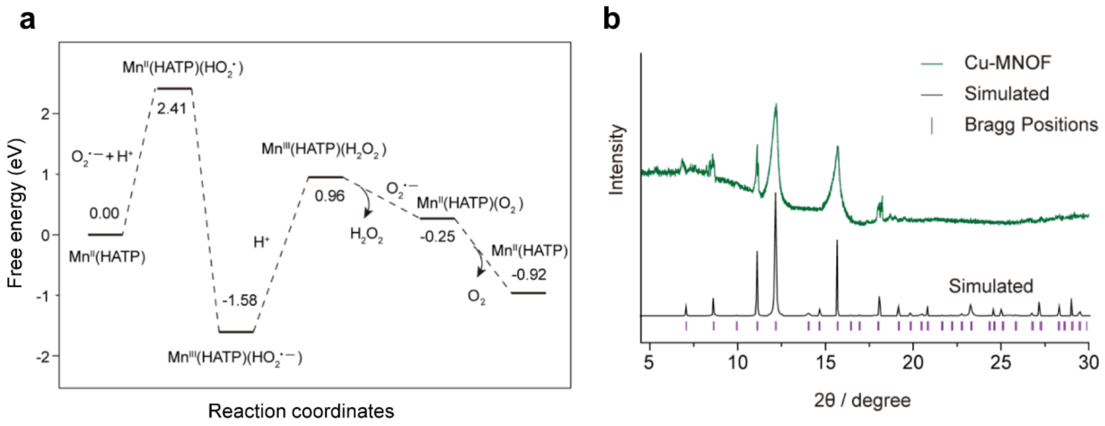

Figure S5. The DFT calculation and PXRD pattern results. (a) Energy profile diagram for MNOF eliminating $O_2^{\bullet-}$. Firstly, the $O_2^{\bullet-}$ captured a hydrogen atom from the H_2O to form $HO_2^{\bullet}$, and then this free radical bond to the Mn center to generate the intermediate state of $Mn^{II}(HATP)(HO_2^{\bullet})$ with the free energy increase of 2.41 eV. Secondly, the intermediate was oxidized to form the $Mn^{III}(HATP)(HOO^{\bullet-})$. Subsequently, this compound was protonated with a hydrogen atom (the free energy increase of 2.54 eV) to release the H_2O_2 molecule. Finally, the second $O_2^{\bullet-}$ bond and transferred an electron to the nanozyme to generate the O_2 molecule, reducing the nanozyme back to the original state. (b) PXRD pattern of Cu-MNOF.

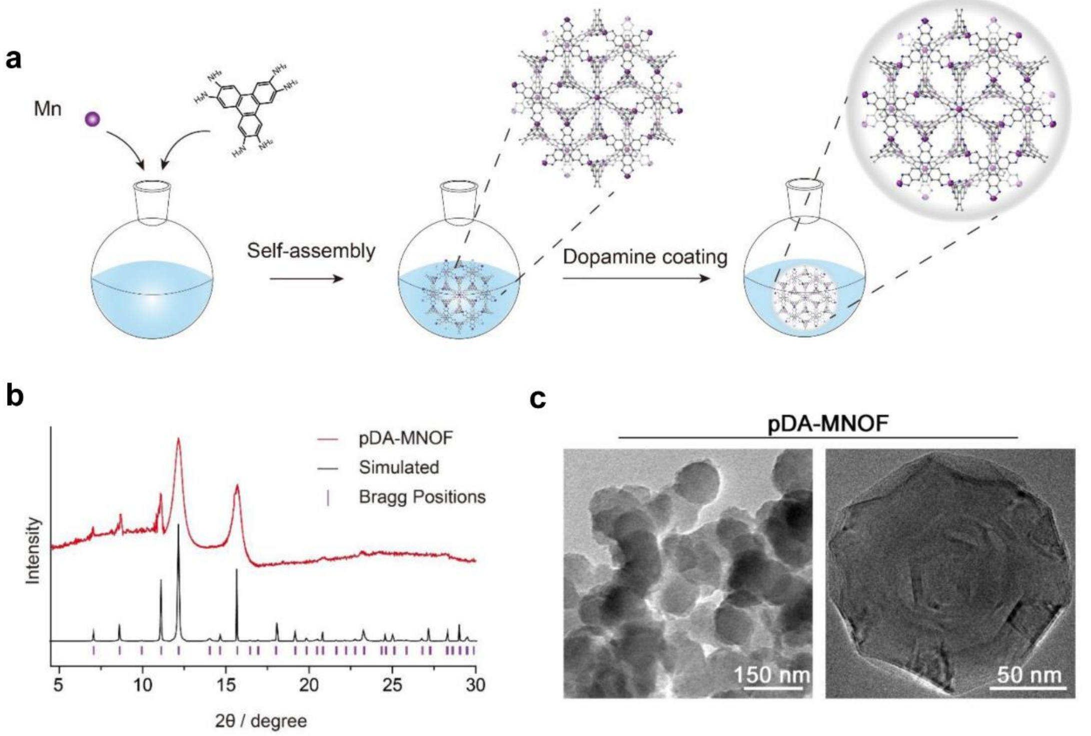

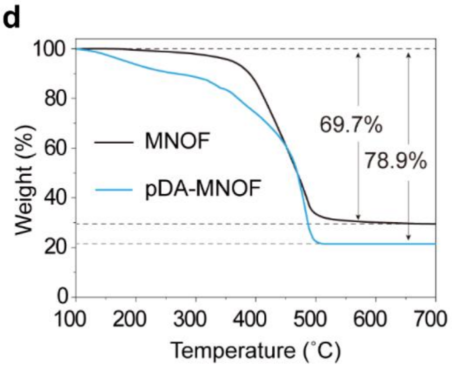

e

	Chemical formula	C	N	H
**MNOF**	$C_{22}O_6N_2H_{20}Mn$	57.02% [a]	6.05% [a]	4.32% [a]
**pDA**	$C_8H_{11}NO_2$	62.75% [a]	9.15% [a]	7.19% [a]
**pDA-MNOF**	N.A.	57.66% [b]	6.34% [b]	4.61% [b]

Figure S6. Fabrication and characterization of pDA-MNOF. (a) Schematic of synthesis of pDA-MNOF. (b) PXRD pattern of the pDA-MNOF (red) and the simulated one (black). (c) The HR-TEM images of pDA-MNOF. (d) TGA analysis of MONF and pDA-MNOF. (e) Elemental analysis of MNOF, pDA and pDA-MNOF. [a], theoretical values; [b], measured values; N.A., not available.

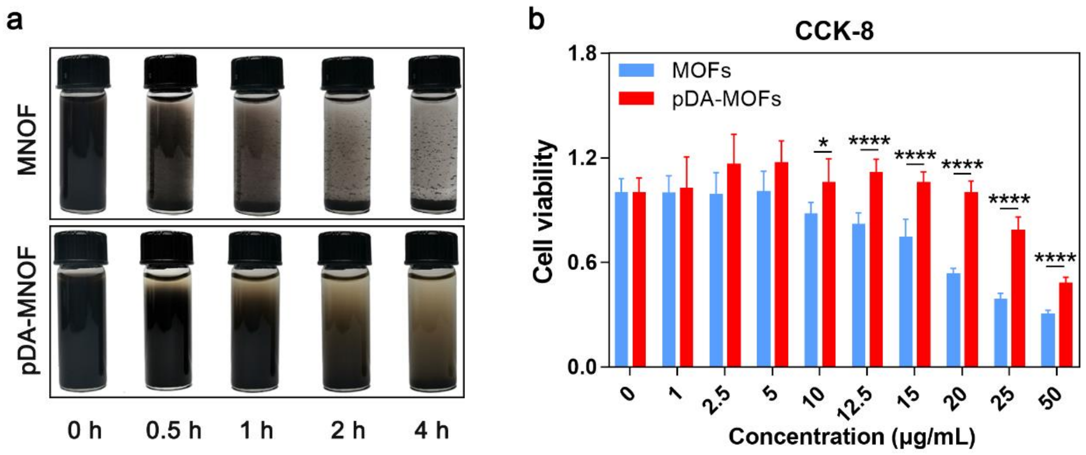

Figure S7. The improved performance of pDA-MNOF on the dispersion stability and cyto-compatibility. (a) Dispersion stability of MNOF and pDA-MNOF in PBS for different times. (b) Cell viability of N2a cells after being treated with various concentrations of MNOF and pDA-MNOF for 24 hours (n = 6). Data were presented with mean ± s.d.; *, P < 0.05; ****, P < 0.0001; T-test.

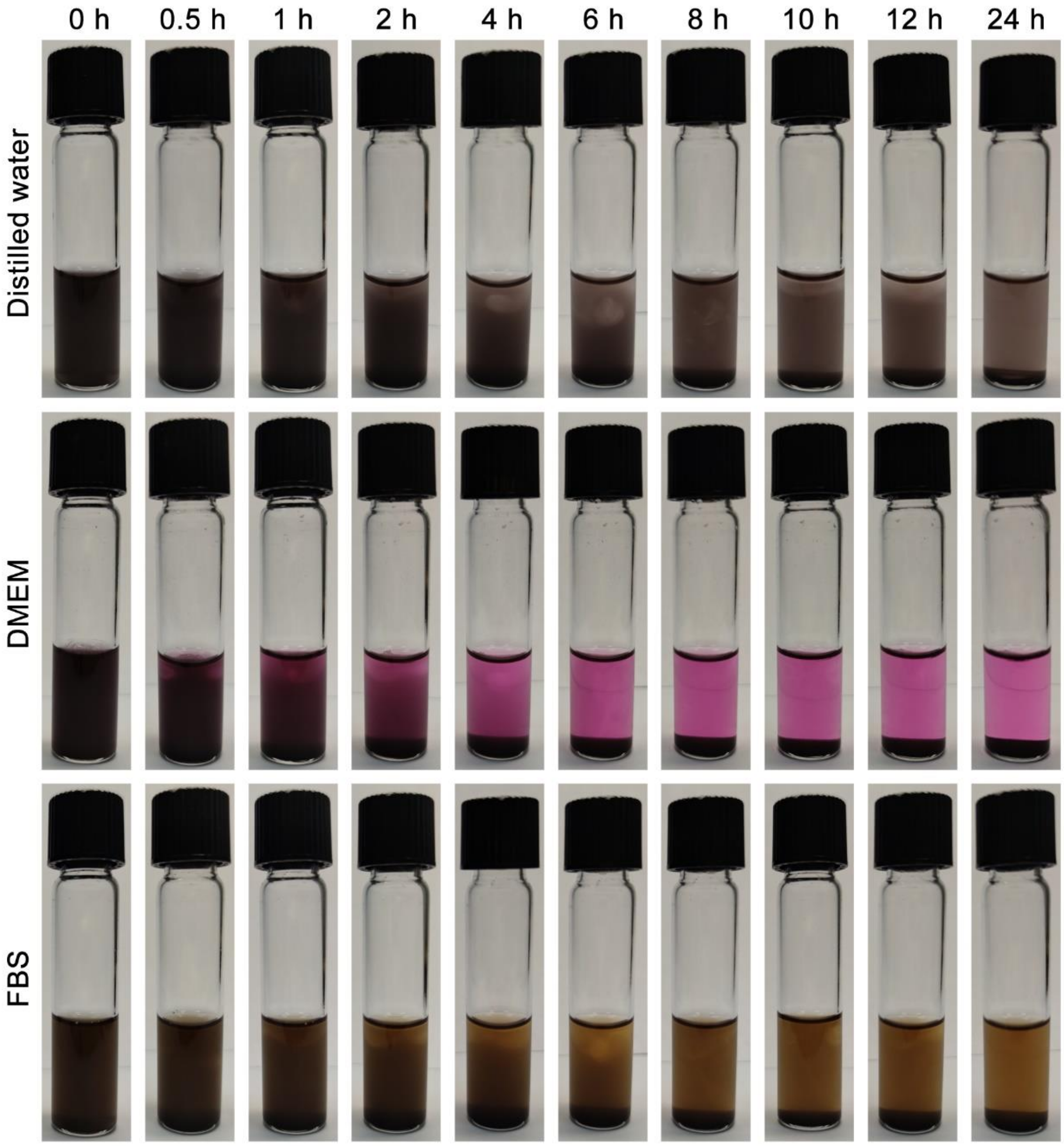

Figure S8. Dispersion stability of MNOF in distilled water, DMEM, and FBS for different times.

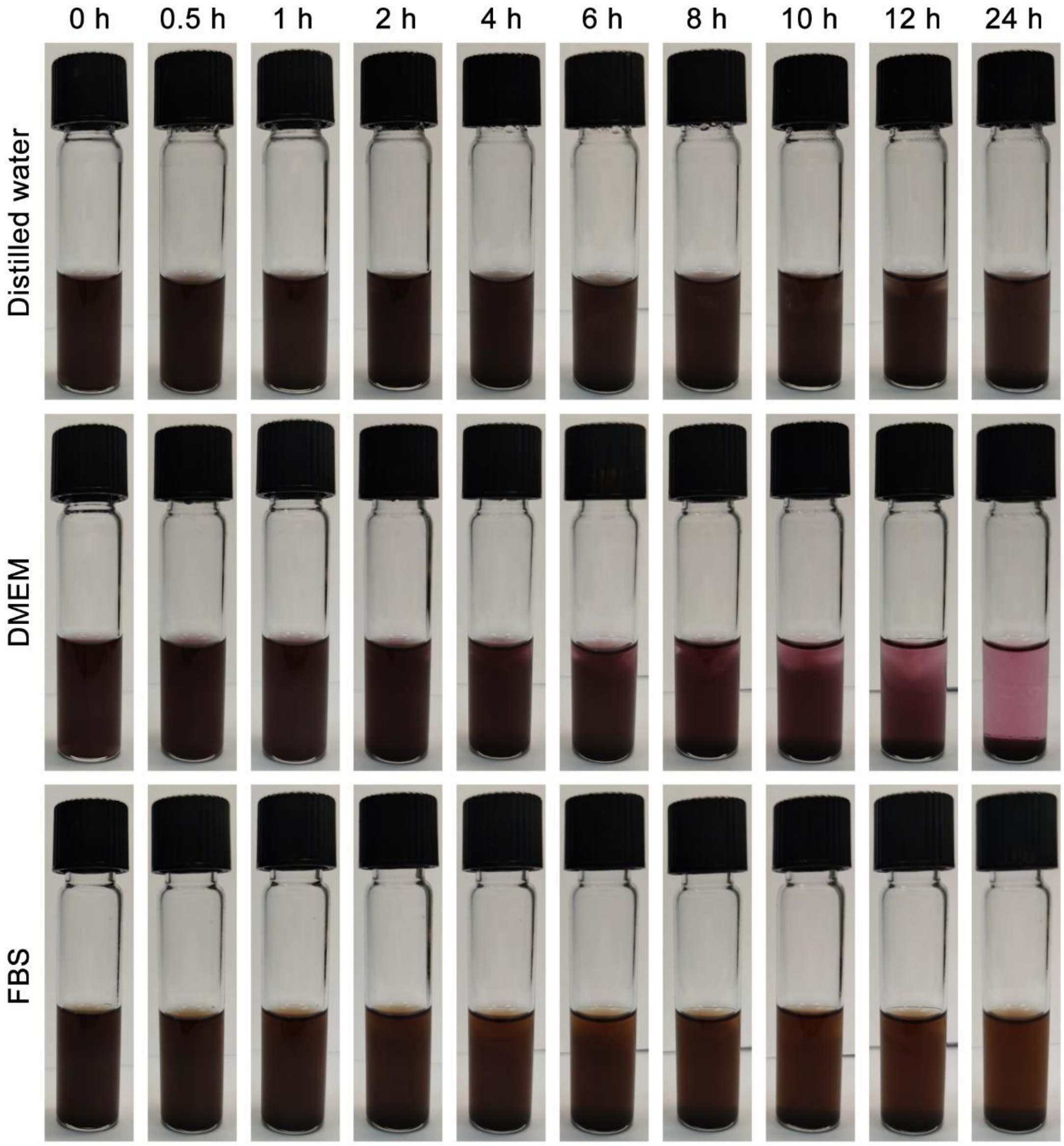

Figure S9. Dispersion stability of pDA-MNOF in distilled water, DMEM, and FBS for different times.

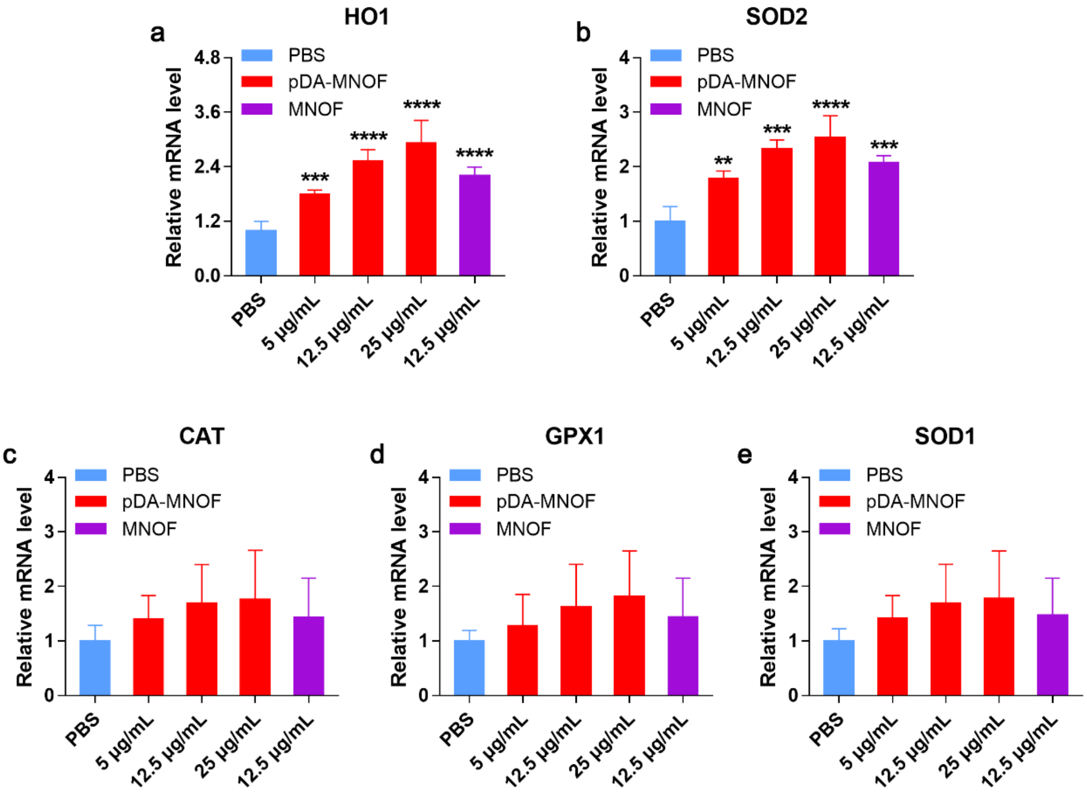

Figure S10. The mRNA levels of anti-oxidative enzymes in pDA-MNOF treated N2a cells. (a-e) The mRNA levels of HO1 (a), SOD2 (b), CAT (c), GPX1 (d) and SOD1 (e) in N2a cells that were treated with PBS, three concentrations of pDA-MNOF and 12.5 μg/mL MNOF for 12 hours ($n = 3$). Data were presented with mean ± s.d.; **, $P < 0.01$; ***, $P < 0.001$; ****, $P < 0.0001$; ANOVA.

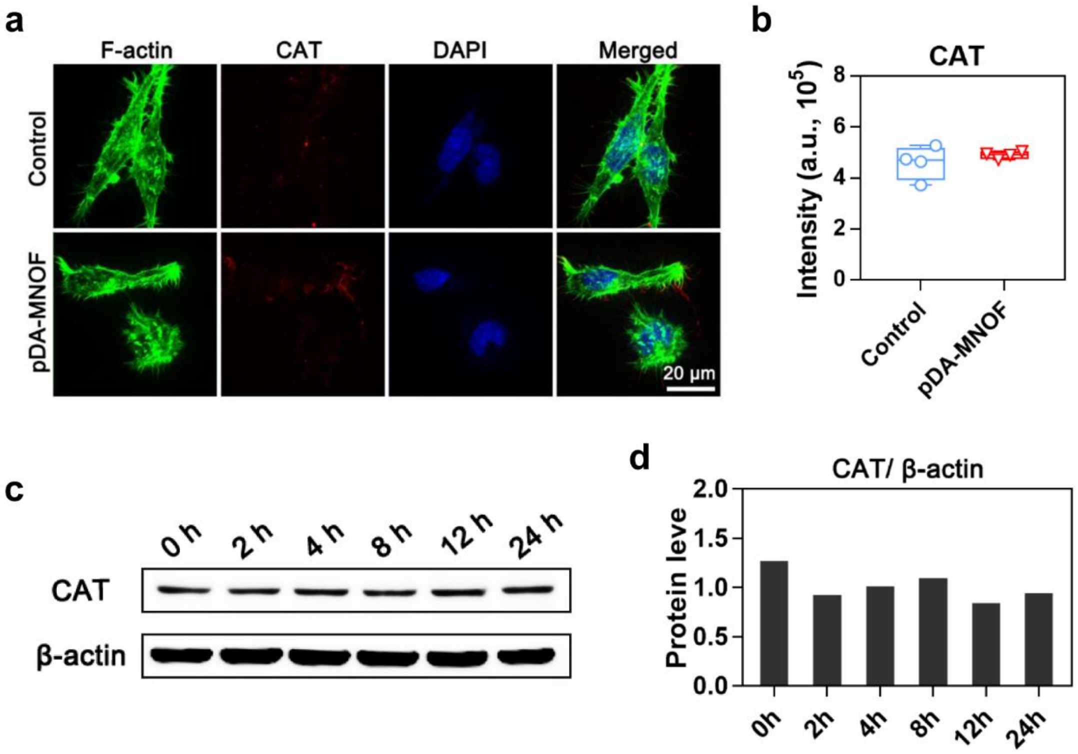

Figure S11. The expression of CAT in pDA-MNOF treated N2a cells. (a) Immunofluorescent staining of F-actin (green), CAT (red) and DAPI (blue) in PBS or 12.5 μg/mL pDA-MNOF treated N2a cells. N2a cells treated with PBS were set as the control. (b) Quantification of fluorescent intensity of CAT in indicated groups in (a) (n = 4). (c) Western blot analysis of CAT and β-actin in N2a cells treated with 12.5 μg/mL pDA-MNOF for different time durations. (d) Quantification of the band intensity ratios of CAT/ β-actin in (c).

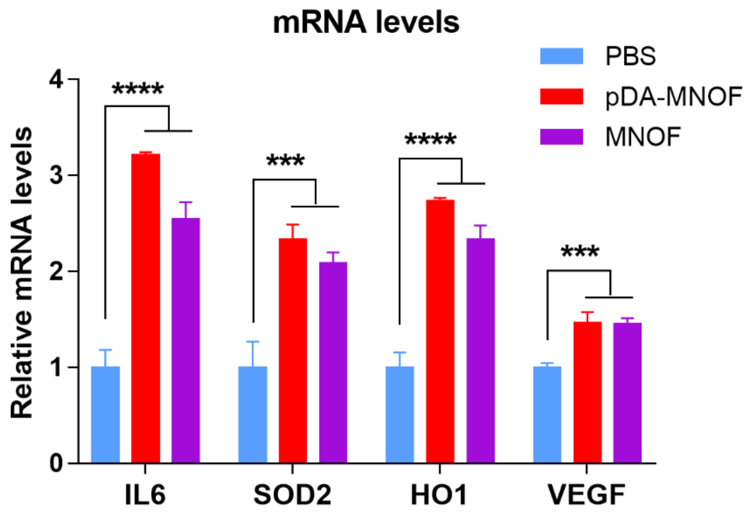

Figure S12. The expression of STAT3-associated genes. The mRNA expression of IL6, SOD2, HO1 and VEGF, in N2a cells treated with PBS, 12.5 μg/mL pDA-MNOF and 12.5 μg/mL MNOF, respectively (n = 3). Data were presented with mean ± s.d.; ***, P < 0.001; ****, P < 0.0001; ANOVA.

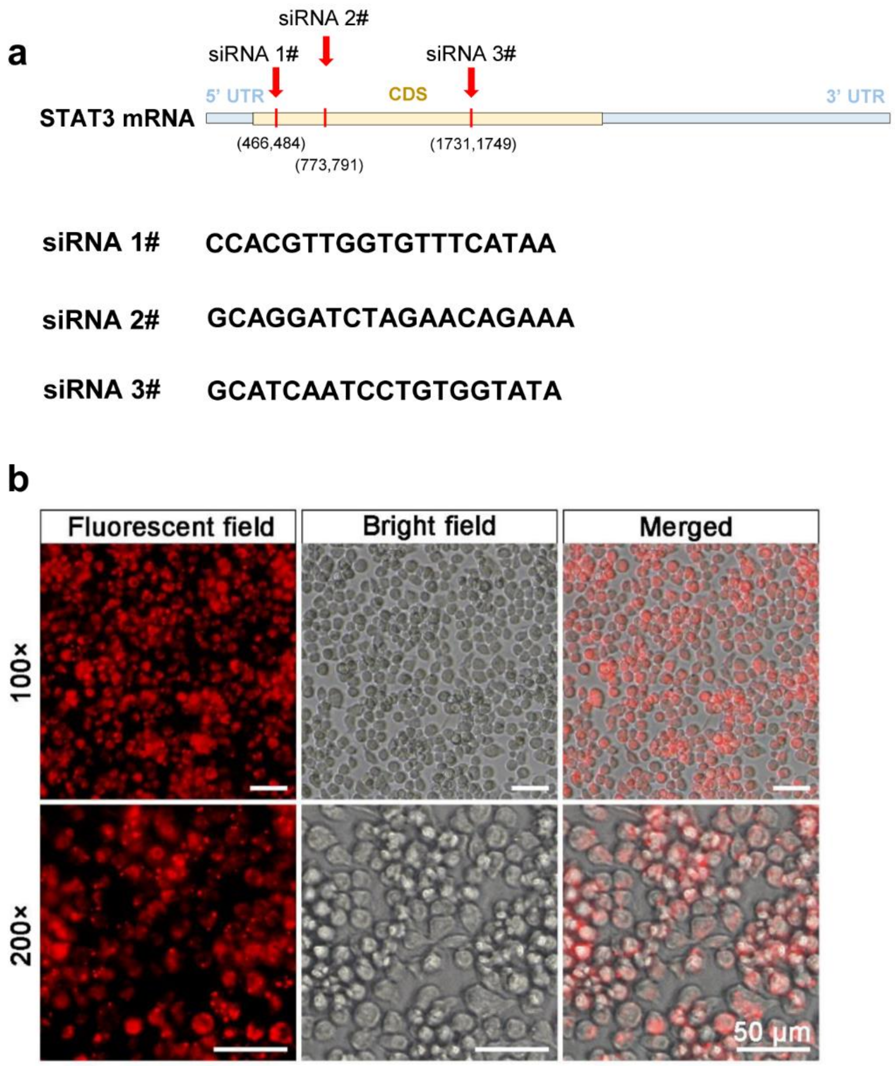

Figure S13. Design of three siRNA targeting STAT3. (a) The siRNAs targeting three sequences of CDS region in STAT3 mRNA were designed and termed as siRNA 1#, siRNA 2#, and siRNA 3#, respectively. The sequences of these siRNA were listed below. (b) The fluorescent images of N2a cells 72 hours after transfected with cy3-labeled siRNA.

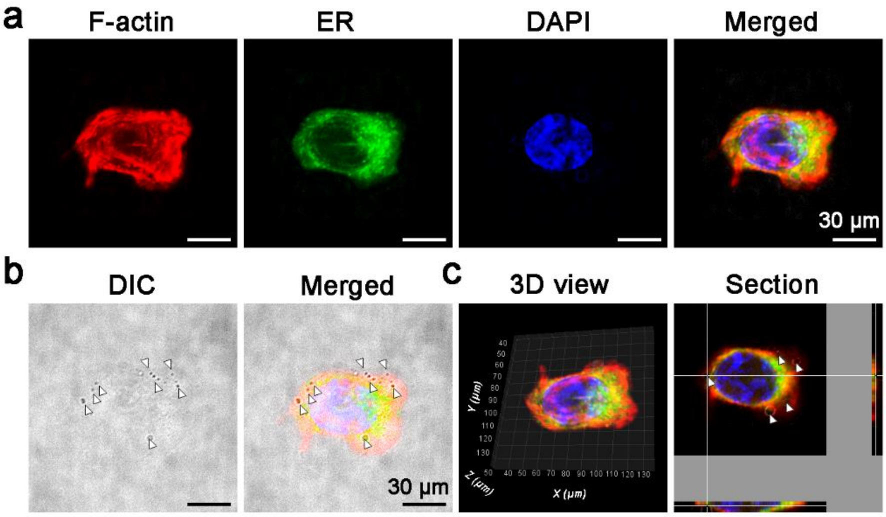

Figure S14. Localization of pDA-MNOF in neuronal cells. (a) Confocal images of N2a cells stained with phalloidin (F-actin, red), ER tracker (ER, green) and DAPI (nuclei, blue) after a 4-h incubation with pDA-MNOF. (b) The DIC and merged images of pDA-MNOF treated N2a cells. These dark granules indicated by white arrowheads were pDA-MNOF. (c) The 3D reconstructed images of the pDA-MNOF treated N2a cells, and the representative image slice from z-stack images showed the localization of this nanozyme within cells. The pDA-MNOF were indicated by white arrowheads.

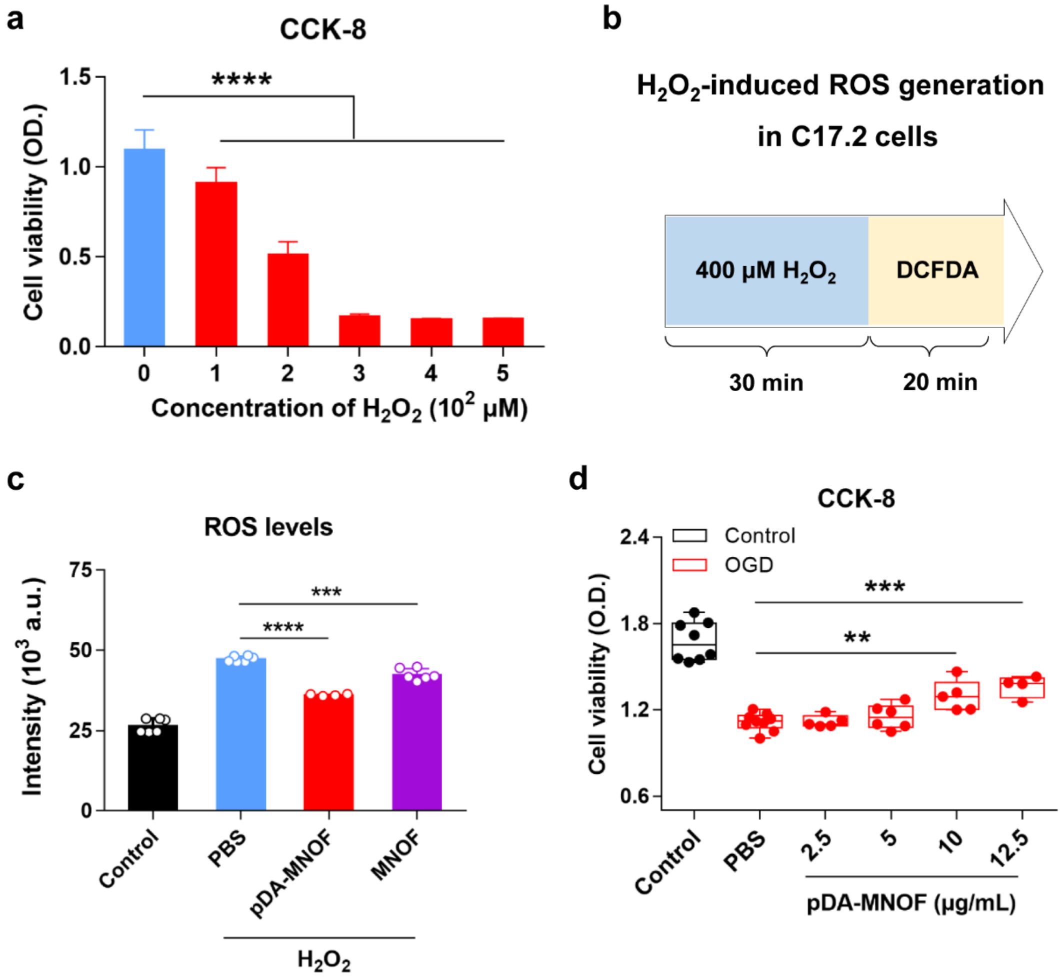

Figure S15. The pDA-MNOF protect C17.2 cells against H_2O_2-induced cell injury. (a) Cell viability of C17.2 cells after treated with various concentrations of H_2O_2 for 24 hours (n = 3). Data were presented with mean ± s.d.; ****, P < 0.0001; ANOVA. (b) Experimental schemes for constructing H_2O_2-induced injury model and testing the cellular ROS. (c) Fluorescence intensity detection in cell lysis of DCFH-DA stained C17.2 cells in indicated groups. Data were presented with mean ± s.d.; ***, P < 0.001; ****, P < 0.0001; ANOVA. (d) Cell viability of C17.2 cells in indicated groups in CCK-8 assay. Data were presented with mean ± s.d.; ** P < 0.01; ***, P < 0.001; ANOVA.

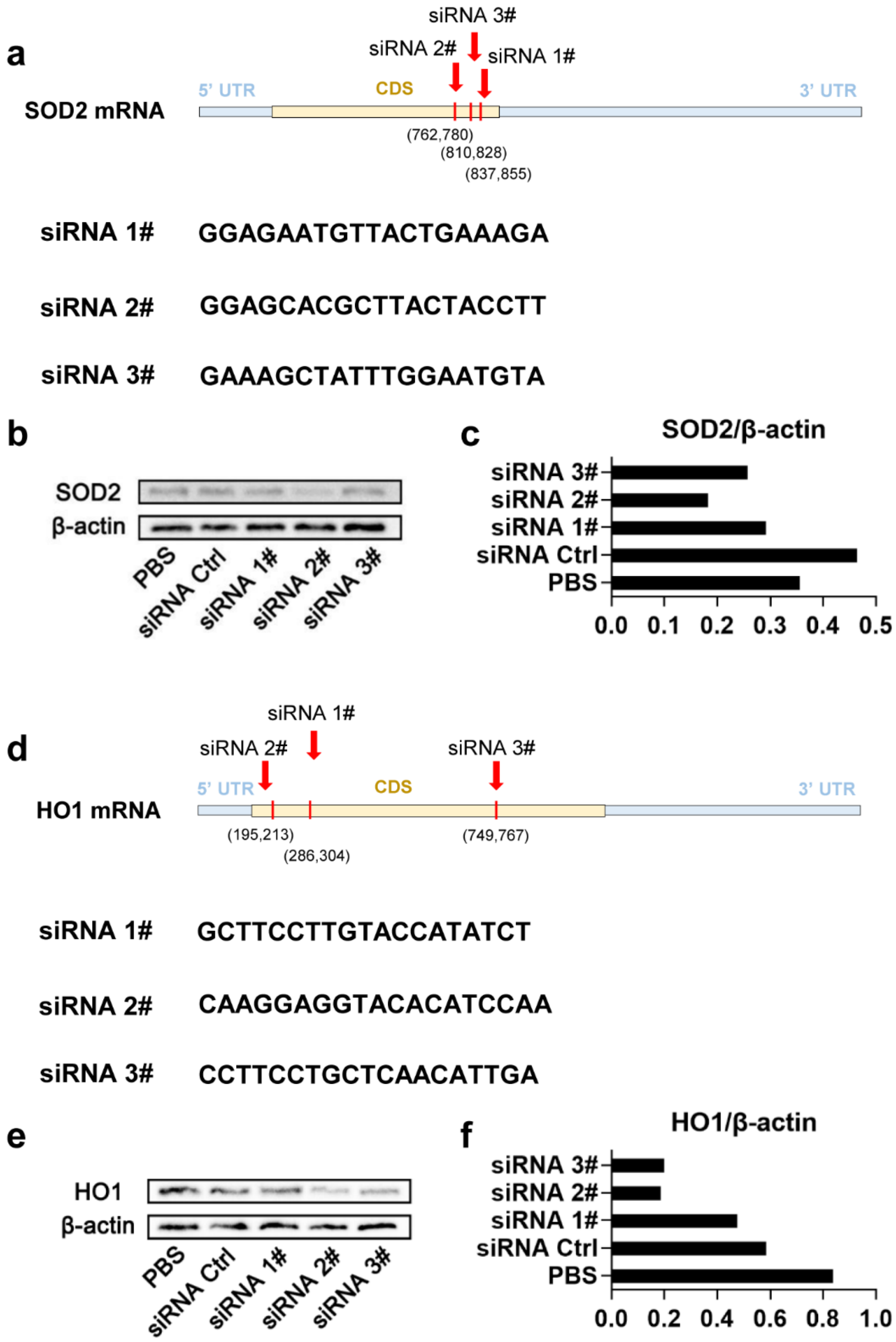

Figure S16. Design and verification of siRNA targeting SOD2 or HO1. (a) Design of three siRNA targeting SOD2. (b) Western blot analysis of SOD2 and β-actin expression in N2a cells treated with PBS, siRNA Ctrl and three siRNAs. (c) Quantification of the band intensity ratios of SOD2/ β-actin in indicated groups in (b). (d) Design of three siRNA targeting HO1. (e) Western blot analysis of HO1 and β-actin expression in N2a cells treated with PBS, siRNA Ctrl and three siRNAs. (f) Quantification of the band intensity ratios of HO1/ β-actin in indicated groups in (e).

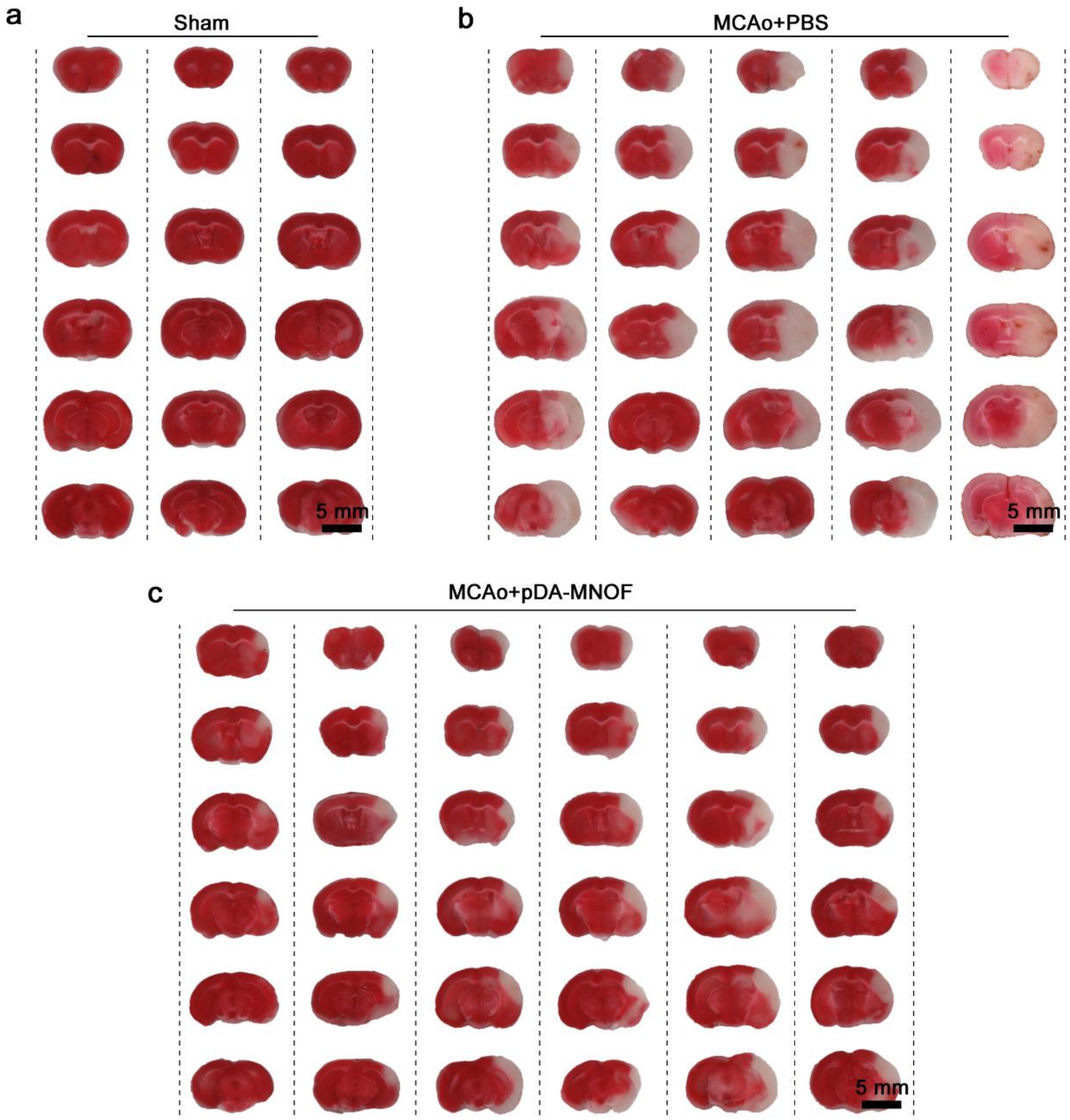

Figure S17. TTC staining for brain sections. (a) Photographs of TTC stained brain sections of mice without suffering MCAo operation. (b-c) TTC staining for brain sections of MCAo mice 24 hours after receiving PBS (b) and pDA-MNOF treatment. Unaffected brain tissues were stained in red and ischemic brain tissues were indicated as white.

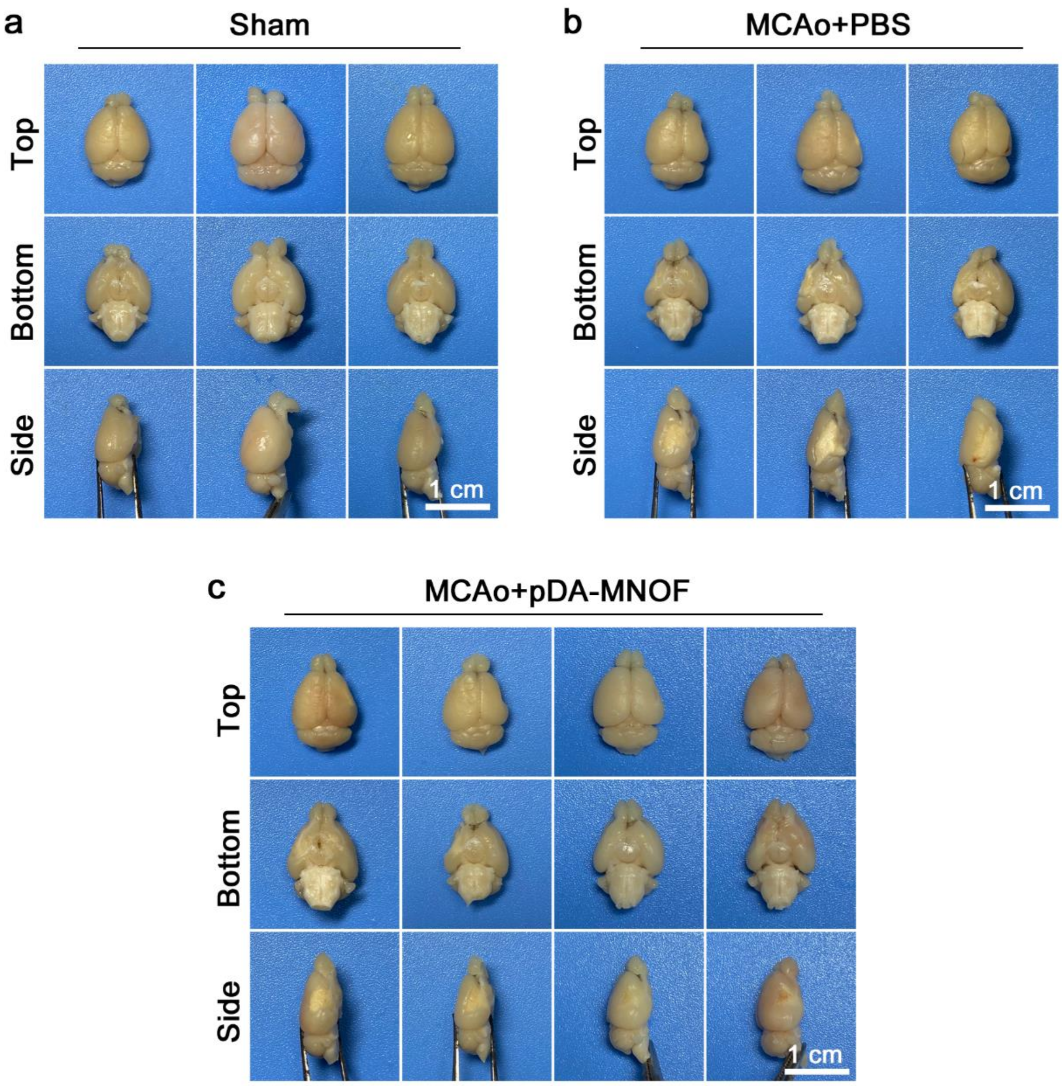

Figure S18. pDA-MNOF reduces the sizes of stroke cavities. (a) Photographs of brains of these mice without suffering MCAo operation (sham) at different views. (b-c) Appearance of brains of these MCAo mice receiving PBS (b) and pDA-MNOF treatment (c) at different views.

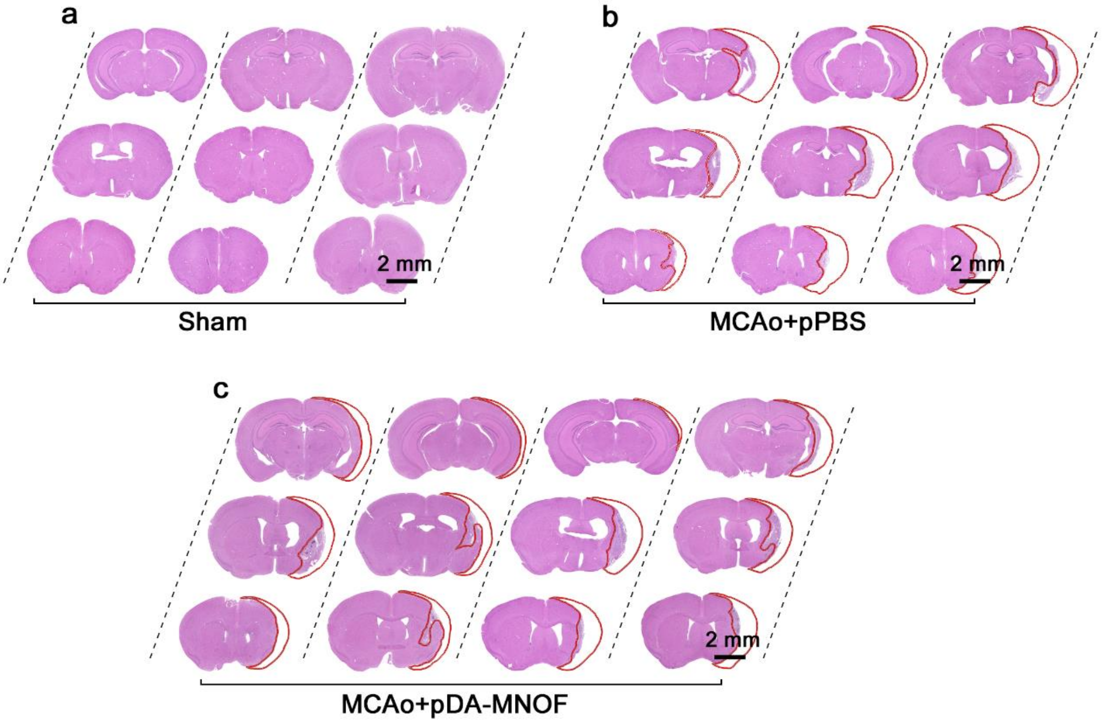

Figure S19. H&E staining for murine brain sections. (a) H&E staining for brain sections of mice without suffering MCAo operation. (b-c) H&E staining for brain section of MCAo mice receiving PBS (b) and pDA-MNOF treatment (c). The missing brain tissues were outlined with red lines.

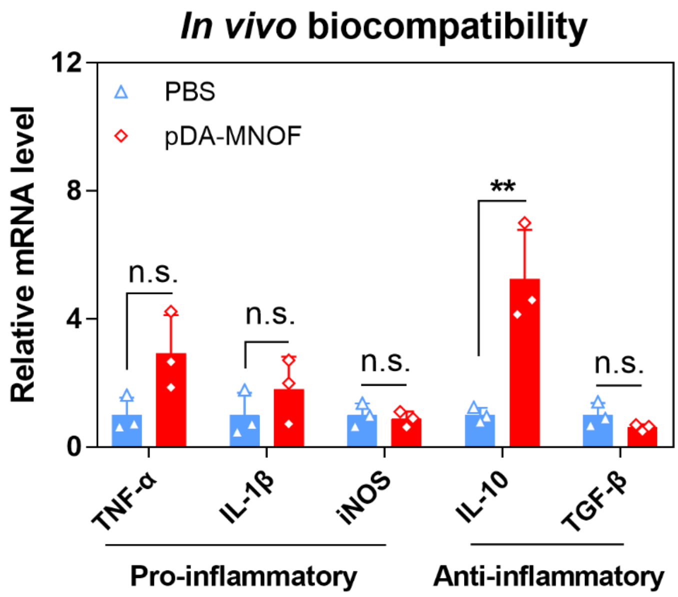

Figure S20. The expression of inflammatory-related cytokine. The mRNA levels of TNF-α, IL-1β, iNOS, IL-10 and TGF-β in the pDA-MNOF treated brain tissues (n = 3). Data were presented with mean ± s.d.; n.s., not significant; **, $P < 0.01$; T-test.

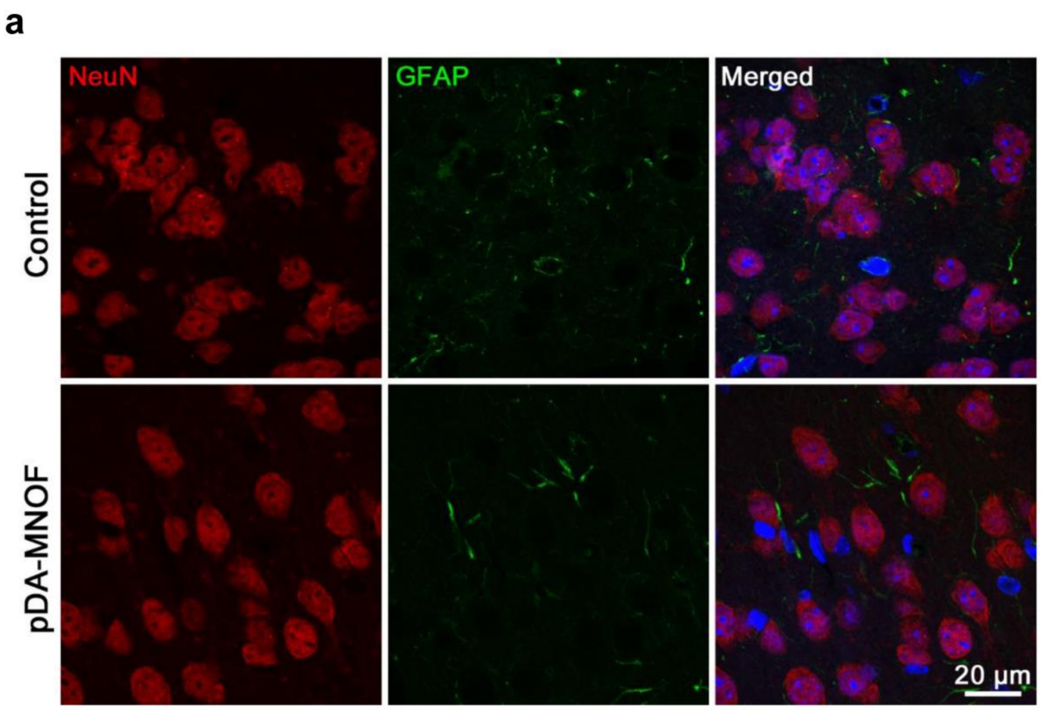

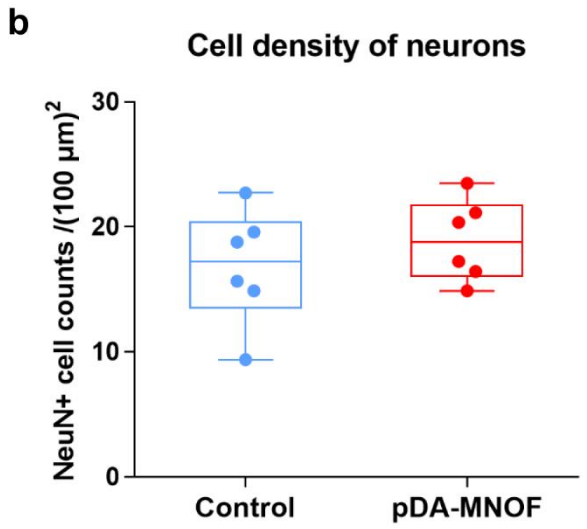

Figure S21. Effects of pDA-MNOF on cell density of neurons *in vivo*. (a) Immunofluorescence staining of NeuN (neurons), GFAP (astrocytes) and DAPI (nuclei) for brain tissues of mice after being receiving cerebral ventricle injection of PBS or pDA-MNOF for 14 days. (b) Quantification of neuron density in indicated groups in (a) (n = 6).

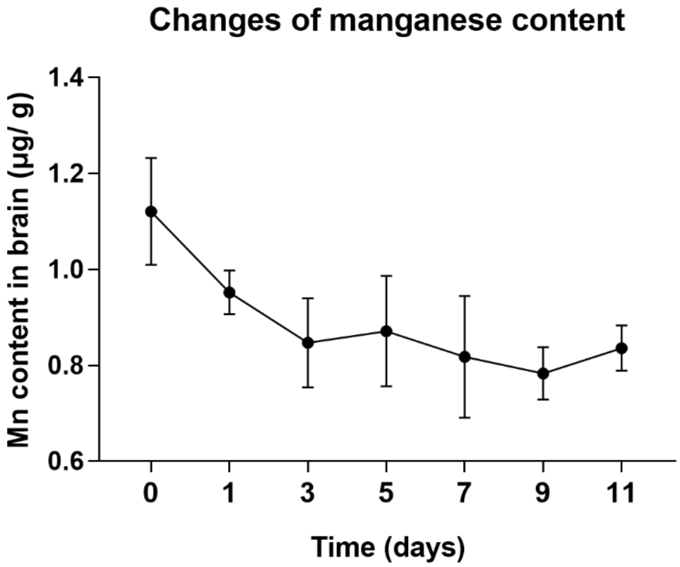

Figure S22. The dynamic changes of manganese content in the brain tissues of MCAo mice receiving pDA-MNOF injection.

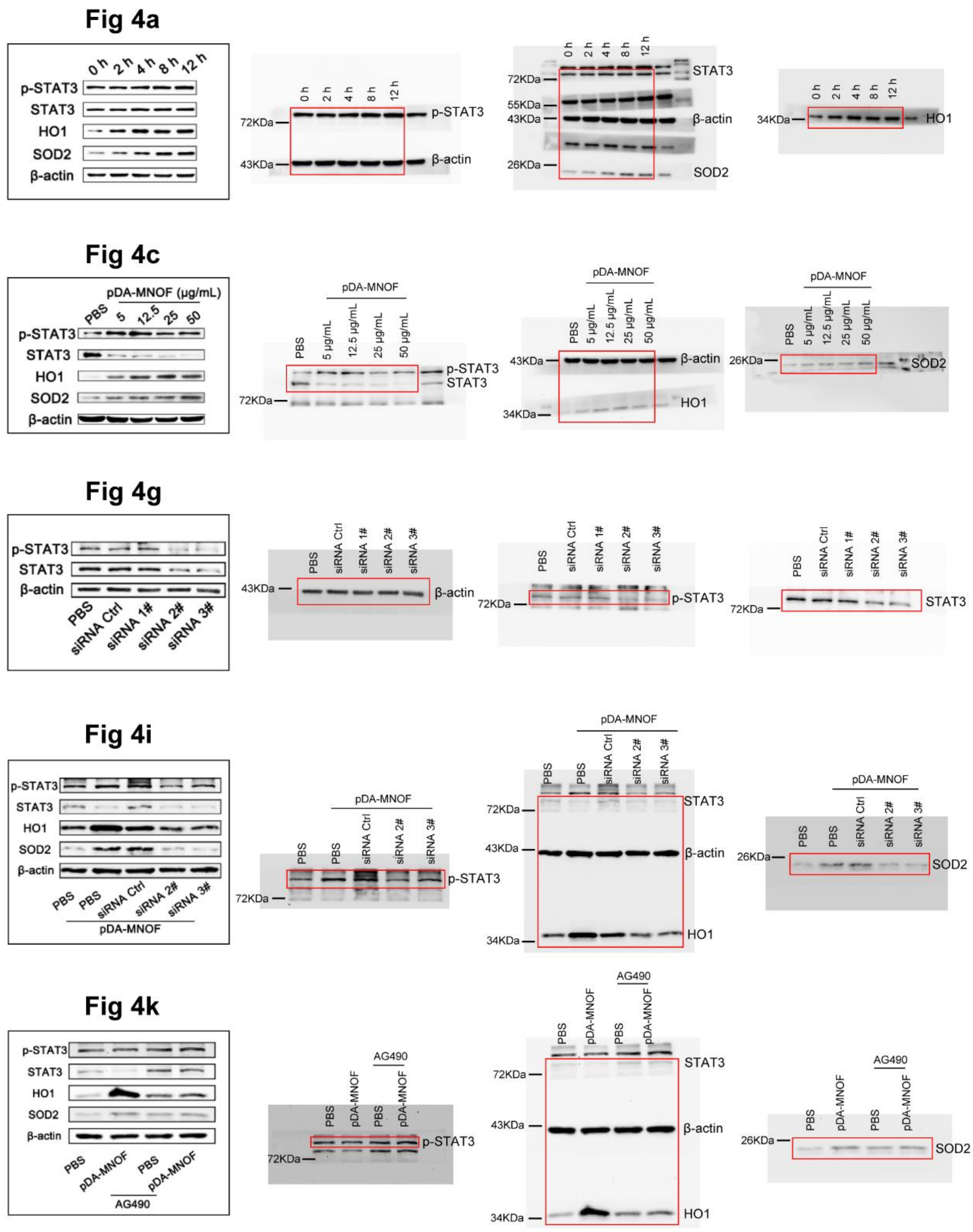

Figure S23. Full western blots for the associated subgraphs of figure 4 in main text.

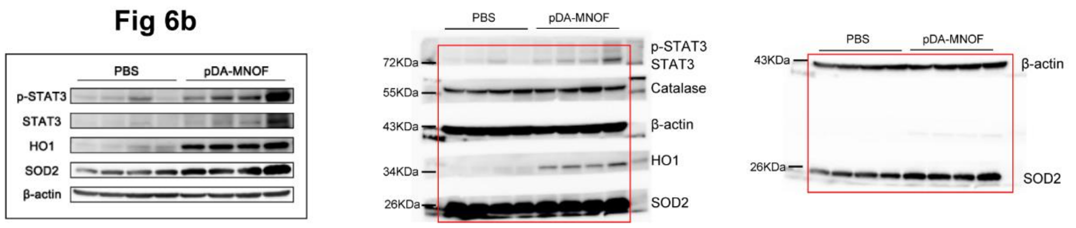

Figure S24. Full western blots for figure 6b in main text.

Table S1. The sequences of qRT-PCR primers.

Genes	Accession No.	Sequences (5'—3')	Length
**HO1**	NM_010442.2	Forward: CCTCACAGATGGCGTCACTT<br>Reverse: GCTGATCTGGGGTTTCCCTC	92 bp
**SOD2**	NM_013671.3	Forward: GTGGGAGTCCAAGGTTCAGG<br>Reverse: TAGTAAGCGTGCTCCCACAC	152 bp
**CAT**	NM_009804.2	Forward: CACTGACGAGATGGCACACT<br>Reverse: TGTGGAGAATCGAACGGCAA	175 bp
**GPX1**	NM_001329527.1	Forward: TCAGTTCGGACACCAGAATGG<br>Reverse: AGGAAGGTAAAGAGCGGGTG	147 bp
**SOD1**	NM_011434.2	Forward: AAGCGGTGAACCAGTTGTGT<br>Reverse: CCAGGTCTCCAACATGCCTC	182 bp
**VEGF**	NM_009505.4	Forward: GGAGTACCCCGACGAGATAG<br>Reverse: CTCCTATGTGCTGGCTTTGGT	167 bp
**TNF-α**	NM_013693.3	Forward: GCACAGAAAGCATGATCCGC<br>Reverse: AACTGATGAGAGGGAGGCCA	213 bp
**IL-1β**	NM_008361.4	Forward: AGCTTCAGGCAGGCAGTATC<br>Reverse: CGTCACACACCAGCAGGTTA	178 bp
**iNOS**	NM_010927.4	Forward: CCCTTCAATGGTTGGTACATGG<br>Reverse: ACATTGATCTCCGTGACAGCC	158 bp
**IL-10**	NM_010548.2	Forward: CAGTACAGCCGGGAAGACAAT<br>Reverse: TTGGCAACCCAAGTAACCCT	180 bp
**TGF-β**	NM_011577.2	Forward: AGGGCTACCATGCCAACTTC<br>Reverse: CCACGTAGTAGACGATGGGC	168 bp
